# EPHA2 mediates PDGFA activity and functions together with PDGFRA as prognostic marker and therapeutic target in glioblastoma

**DOI:** 10.1038/s41392-021-00855-2

**Published:** 2022-02-02

**Authors:** Qu-Jing Gai, Zhen Fu, Jiang He, Min Mao, Xiao-Xue Yao, Yan Qin, Xi Lan, Lin Zhang, Jing-Ya Miao, Yan-Xia Wang, Jiang Zhu, Fei-Cheng Yang, Hui-Min Lu, Ze-Xuan Yan, Fang-Lin Chen, Yu Shi, Yi-Fang Ping, You-Hong Cui, Xia Zhang, Xindong Liu, Xiao-Hong Yao, Sheng-Qing Lv, Xiu-Wu Bian, Yan Wang

**Affiliations:** 1 Institute of Pathology and Southwest Cancer Center, Southwest Hospital, Army Medical University (Third Military Medical University), Chongqing, China; 2 Biobank of Institute of Pathology, Southwest Hospital, Army Medical University (Third Military Medical University), Chongqing, China; 3 Institute of Cancer, Xinqiao Hospital, Army Medical University (Third Military Medical University), Chongqing, China; 4 Department of Neurosurgery, Xinqiao Hospital, Army Medical University (Third Military Medical University), Chongqing, China

**Keywords:** CNS cancer, CNS cancer

## Abstract

Platelet-derived growth subunit A (PDGFA) plays critical roles in development of glioblastoma (GBM) with substantial evidence from TCGA database analyses and in vivo mouse models. So far, only platelet-derived growth receptor α (PDGFRA) has been identified as receptor for PDGFA. However, PDGFA and PDGFRA are categorized into different molecular subtypes of GBM in TCGA_GBM database. Our data herein further showed that activity or expression deficiency of PDGFRA did not effectively block PDGFA activity. Therefore, PDGFRA might be not necessary for PDGFA function.To profile proteins involved in PDGFA function, we performed co-immunoprecipitation (Co-IP) and Mass Spectrum (MS) and delineated the network of PDGFA-associated proteins for the first time. Unexpectedly, the data showed that EPHA2 could be temporally activated by PDGFA even without activation of PDGFRA and AKT. Furthermore, MS, Co-IP, in vitro binding thermodynamics, and proximity ligation assay consistently proved the interaction of EPHA2 and PDGFA. In addition, we observed that high expression of EPHA2 leaded to upregulation of PDGF signaling targets in TCGA_GBM database and clinical GBM samples. Co-upregulation of PDGFRA and EPHA2 leaded to worse patient prognosis and poorer therapeutic effects than other contexts, which might arise from expression elevation of genes related with malignant molecular subtypes and invasive growth. Due to PDGFA-induced EPHA2 activation, blocking PDGFRA by inhibitor could not effectively suppress proliferation of GBM cells, but simultaneous inhibition of both EPHA2 and PDGFRA showed synergetic inhibitory effects on GBM cells in vitro and in vivo. Taken together, our study provided new insights on PDGFA function and revealed EPHA2 as a potential receptor of PDGFA. EPHA2 might contribute to PDGFA signaling transduction in combination with PDGFRA and mediate the resistance of GBM cells to PDGFRA inhibitor. Therefore, combination of inhibitors targeting PDGFRA and EHA2 represented a promising therapeutic strategy for GBM treatment.

## Introduction

Glioma is the most prevalent brain tumor and pathologically categorized into four grades (I–IV) by the 2016 World Health Organization (WHO) classification of central nervous system tumors.^1^ Grade I and II gliomas are considered as low-grade glioma (LGG), assuming slow progression and favorable prognosis, but grade III and IV gliomas are high-grade glioma (HGG) and featured with highly invasive growth and significantly shortened survival.^2–6^ Grade IV glioma, also known as glioblastoma multiforme (GBM), is the most malignant form of glioma and remains intractable despite the progression of surgical and pharmacological therapies. The average survival time of GBM patients is about 15 months and the 5-year survival rate is less than 5%.^7–11^ In clinic, majority of GBM patients (about 90%) are diagnosed with wild-type IDH1/2 and have no glioma history, which is defined as primary or de novo GBM. About 10% GBM patients harbor IDH1/2 mutation and have a history of lower-grade glioma, which is defined as secondary GBM.^1,12,13^ The Cancer Genome Atlas (TCGA) project have unveiled comprehensive genetic and transcriptomic profiles of GBM through next-generation sequencing,^14^ which classifies GBM into four molecular subtypes: Proneural, Neural, Mesenchymal, and Classical.^15^

Platelet-derived growth factor receptor α (PDGFRA) and β (PDGFRB) belong to receptor tyrosine kinase (RTK) family and function as receptors for platelet-derived growth factor (PDGF). Four PDGF genes (PDGFA, PDGFB, PDGFC, and PDGFD) in mammalian have been identified and encode four peptides (PDGFA, PDGFB, PDGFC, and PDGFD), which form five functional homo- or hetero-dimers: PDGF-AA, PDGF-AB, PDGF-BB, PDGF-CC, and PDGF-DD. When stimulated with dimeric PDGF peptides, PDGFRA and PDGFRB immediately form homo- or hetero-dimer and undergo autophosphorylation for full activation. Activated PDGF/PDGFR axis continue to activate downstream signaling pathways, such as PI3K/AKT/mTOR pathway, RAS/MAPK/ERK pathway, and JAK/STAT3 pathway, which lead to proliferation, survival, and invasion of cancer cells.^16,17^ Specificity of interactions between PDGF ligands and PDGFR receptors have been clarified: PDGFRA homodimer are activated by PDGF-AA, -AB, and -CC, PDGFRB homodimer are activated by PDGF-BB and -DD, and PDGFRA/PDGFRB heterodimer are only activated by PDGF-AB.^16,17^ Therefore, PDGFRA is the only identified receptor to mediatePDGFA function in GBM.

Both PDGFA and PDGFRA have been found to play critical roles in gliomagenesis and tumor progression.^15,18–20^ On the one hand, PDGFA is one of the core genes related with gain of chromosome 7, which is broadly observed in glioblastoma,^18,21^ and also one of the signature genes for the classical subtype GBM^15^; on the other hand, copy number amplification and mRNA overexpression of *PDGFRA* are typical features of Proneural GBM. Experimentally, overexpression of PDGFA and PDGFRA successfully induces GBM development in mouse models.^22–25^ These results suggest critical roles of PDGFRA in GBM and identify PDGFA/PDGFRA axis as a potential therapeutic target for GBM.^16^ Indeed, several anti-tumor agents targeting PDGFRA have been developed, such as Imatinib (Gleevec^®^), Sorafenib (Nexavar^®^), Nilotinib (Tasigna^®^), and Sunitinib (Sutent^®^). Although the data in vitro and in vivo support the potent inhibitory effects of targeting PDGFRA in GBM cells,^26,27^ clinical trials of single PDGFRA inhibitor have failed to show anti-tumor effects.^28,29^ Therefore, the regulation mechanisms on PDGFA and PDGFRA in GBM need be clarified before clinical application of strategies targeting PDGFA/PDGFRA signaling axis. In this work, through co-immunoprecipitation (Co-IP) and Mass-Spectrum (MS) identification, we profiled PDGFA-associated proteins and revealed EPHA2 as a new receptor for PDGFA to mediate PDGFA function even without PDGFRA.

## Results

### PDGFRA was not necessary for PDGFA signaling in GBM

**Fig. 1:**
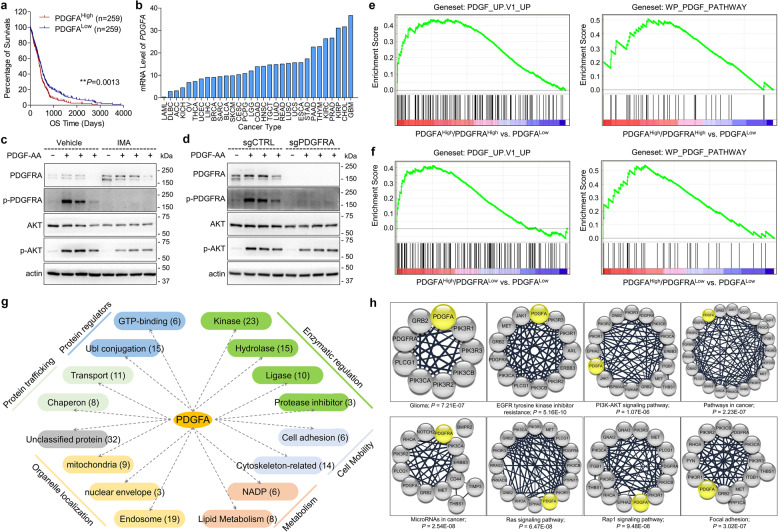
PDGFRA is not necessary for PDGFA function in GBM cells. **a** Kaplan–Meier survival analysis of cases with PDGFA^High^ *vs*. PDGFA^Low^ from TCGA_GBM database. **b** PDGFA gene expression in TCGA PanCancer databases. **c** PDGFA-induced temporal expression of indicated proteins in LN18 cells pre-treated with vehicle or IMA. β-actin is used as loading control. **d** PDGFA-induced temporal expression of indicated proteins in LN18 cells transfected with control CRISPR/Cas9 or CRISPR/Cas9 targeting PDGFRA. **e**, **f** Enrichment of PDGF signaling-related genesets for TCGA_GBM cases with PDGFA^High^/PDGFRA^High^ vs. PDGFA^Low^ or PDGFA^High^/PDGFRA^Low^ vs. PDGFA^Low^. **g** Categorization of PDGFA-associated proteins. **h** Significantly enriched KEGG pathways involving PDGFA through KEGG analysis on PDGFA interactome.

First, we analyzed four known PDGF genes in TCGA_GBM database through Kaplan–Meier survival analysis. The result indicated that high expression of PDGFA predicted poor survival of GBM patients (Fig. 1a) but the other three PDGF ligands did not show such prognostic significance (Supplementary Fig. S1a). In addition, gene expression comparison among various types of cancers in TCGA PanCan databases showed that GBM held the highest mRNA level of PDGFA (Fig. 1b), but not the other three PDGF ligands (supplementary Fig. S1b), implying that PDGFA was particularly important for GBM. Since only PDGFRA has been identified as the receptor for PDGFA, we examined PDGFRA protein in a panel of glioma cells, including 2 primary GBM cells (091214 and 090116), 7 commercial GBM cell lines (A172, DBTRG-05MG, LN18, LN229, T98G, U251, and U87), and 1 commercial grade III glioma cell line (SW1088). Western blotting results showed that PDGFRA protein was detectable in 091214, LN18, SW1088, and U251 cells (Supplementary Fig. S1c). Since PDGFRA was not detected in LN229 cell line, we constructed a PDGFRA-overexpression cell line using LN229 (LN229^PDGFRA^) to examine endogenous PDGFRA functions from other cell lines (Supplementary Fig. S1d). Our data showed that recombinant human PDGF-AA (100 ng/ml)^30^ stimulation resulted in phosphorylation of PDGFRA without regulation on PDGFRB (Supplementary Fig. S1e). However, PDGF-BB leaded to activation of both PDGFRA and PDGFRB (Supplementary Fig. S1e). To evaluate the effects of inhibitors on PDGFRA on PDGFA signaling, we pre-treated GBM cells with imatinib (IMA), a potent PDGFRA inhibitor, but we surprisingly found that inhibition on PDGFRA activity could not diminish the activation of AKT, a well-known downstream target of PDGFA (Fig. 1c). In accordance with this observation, depletion of PDGFRA in LN18 cells (LN18^PDGFRA-/-^) through CRISPR/Cas9 technology did not completely block PDGFA signaling (Fig. 1d). Interestingly, analysis on TCGA_GBM and TCGA_GBMLGG databases showed that PDGFA and PDGFRA were negatively correlated with each other and their expression patterns in various molecular subtypes were also inconsistent (Supplementary Fig. S1f and S1g). Then, we classified 539 GBM cases of TCGA_GBM according to gene expression levels of PDGFA and PDGFRA into Subgroup 1 (PDGFA^High^/PDGFRA^High^, *n* = 25), Subgroup 2 (PDGFA^High^/PDGFRA^Low^, *n* = 35), and Subgroup 3 (PDGFA^Low^, *n* = 67) (Dataset 1). As expected, geneset enrichment assay (GSEA)^31,32^ showed that Subgroup 1 significantly enriched genesets of PDGF_UP.V1_UP (M2834 from Molecular Signatures Database v7.4) (ES = 0.4392; *P* = 0.008) and WP_PDGF_PATHWAY (M39555 from Molecular Signatures Database v7.4) (ES = 0.5076; *P* = 0.0403) compared to Subgroup 3 (Fig. 1e). Intriguingly, Subgroup 2 significantly enriched genesets of PDGF_UP.V1_UP (ES = 0.4125: *P* = 0.0049) and WP_PDGF_PATHWAY (ES = 0.5424: *P* = 0.008) compared to Subgroup 3 (Fig. 1f). However, PDGF_UP.V1_UP and WP_PDGF_PATHWAY genesets was not enriched by Subgroup 1 *versus* Subgroup 2 (*P* = 0.2455 and *P* = 0.9876, respectively) (Data not shown). These data implied that the expression level of PDGFRA was not decisive for PDGFA signaling and PDGFA might function in a PDGFRA-independent manner in GBM cells.

To understand the regulation network of PDGFA in GBM cells, we profiled PDGFA interactome using LN18 cells. For this purpose, the LN18 cells were treated with 100 ng/ml recombinant human PDGF-AA for 15 min and harvested for Co-IP with anti-PDGFA antibody. Three separated Co-IP samples (triplicate) were subjected to MS identification and 189 proteins were consistently detected including PDGFA itself (Dataset 2). According to the protein functional keywords, 157 of 189 proteins could be categorized into six groups, including enzymatic regulation, protein regulator, protein trafficking, cell mobility, metabolism, and organelle localization (Fig. 1g and Supplementary Table S1). Then, we analyzed the interactome via DAVID Bioinformatics Resources 6.8 (https://david.ncifcrf.gov/). KEGG pathway analysis indicated that the PDGFA-associated proteins were mainly cancer and neural system-related (Supplementary Fig. S1h), such as proteoglycans in cancer, pathways in cancer, glioma, and axon guidance. Moreover, in KEGG pathways, top-ranked eight PDGFA-involved categories according to *P*-Value were EGFR tyrosine kinase inhibitor resistance, Ras signaling pathway, Pathways in cancer, Glioma, MicroRNAs in cancer, Rap1 signaling pathway, focal adhesion, and PI3K-AKT signaling pathway (Fig. 1h). GO analysis showed that PDGFA-associated proteins played important roles in PI3K activity, tyrosine kinase activity, ubiquitin ligase activity, and protein transport (Supplementary Fig. S1i). In the kinase category of PDGFA interactome, PDGFRA, PIK3R1, PIK3R2, PIK3R3, PIK3CA, and PIK3CB were highly abundant (Dataset 2 and Supplementary Table S1). Since PDGFA strongly activated PI3K/AKT pathway through PDGFRA, the interaction of PDGFA with these proteins confirmed the reliability of PDGFA interactome. Furthermore, we noticed that the kinases consistently contained SH2-domain (Supplementary Fig. S1j), which are known to be responsible for RTK activity and trafficking. Together, our study profiled the regulation network of PDGFA and emphasized the critical implication of a series of kinases.

### EPHA2 was activated by PDGFA in a PDGFRA-independent manner

**Fig. 2:**
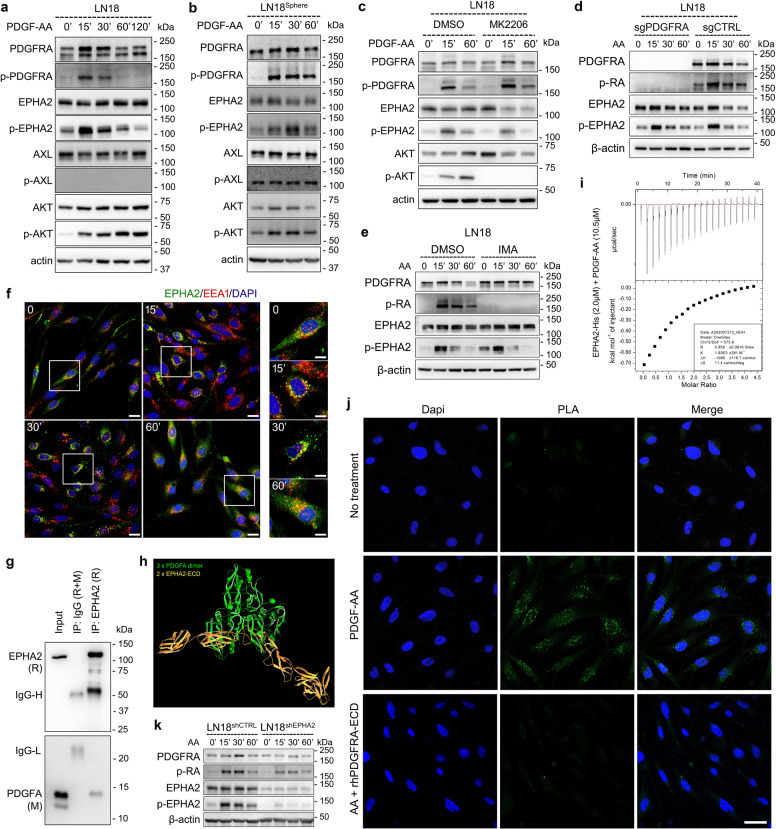
PDGFA activates EPHA2 in a PDGFRA-independent manner in GBM cells. **a** PDGFA-induced temporal expression of indicated proteins in LN18 cells examined by western blotting. β-actin is used as loading control. **b** PDGFA-induced temporal expression of indicated proteins in LN18 Sphere examined by western blotting. **c** PDGF-A-induced temporal expression of indicated proteins in LN18 cells pre-treated with DMSO and MK2206. **d** PDGFA-induced temporal expression of indicated proteins in LN18 cells infected with lentivirus containing control sgRNA or sgRNA targeting PDGFRA. **e** PDGFA-induced temporal expression of indicated proteins in LN18 cells pre-treated with vehicle or IMA. **f** Representative immunofluorescence images stained by antibodies targeting EPHA2 and EEA1, respectively. DAPI is used to label nuclei. Scale bar = 10 μm for large four panels and 5 μm for small four panels. **g** Co-immunoprecipitation and western blotting of EPHA2 in PDGFA-treated LN18 cells. **h** Interaction simulation of three-dimension structure of PDGFA and EPHA2 extracellular domain. **i** Interaction thermodynamics of recombinant human EPHA2 extracellular domain and recombinant human PDGF-AA using Microcal iTC200. **j** Proximity ligation assay using LN18 cells without treatment, treated with PDGFA for 15 min, or pre-treated with recombinant PDGFRA extracellular domain followed by PDGFA treatment for 15 min. The cells is counterstained with Dapi (blue) to mark nuclei. Green dot signals represent interaction between EPHA2 and PDGFA. Scale Bar = 25 μm. **k** PDGFA-induced temporal expression of indicated proteins in LN18 cells infected with lentivirus containing control shRNA or shRNA targeting EPHA2.

In the Kinase category, we noticed two RTKs, EPHA2 and AXL, with high abundance (Dataset 2). Interestingly, treatment of PDGF-AA dramatically led to EPHA2 phosphorylation but not AXL in GBM cell lines with endogenous or exogenous PDGFRA expression (Fig. 2a and Supplementary Fig. S2a). We cultured LN18 GBM cell line as sphere and similarly observed PDGF-AA-induced EPHA2 activation (Fig. 2b and Supplementary Fig. S2b). Since EPHA2 has been reported to be phosphorylated by activated AKT, we blocked the activation of AKT by MK2206, an AKT inhibitor. Although the AKT activation was completely blocked by the inhibitor, PDGF-AA-induced EPHA2 phosphorylation was not significantly affected (Fig. 2c and Supplementary Fig. S2c). It was noted that EPHA2 phosphorylation showed a consistent pattern with phosphorylated PDGFRA, which promoted us to investigate if the phosphorylation of EPHA2 happened parallelly with or as a result of PDGFRA activation. For this purpose, we treated parental LN18 and LN18^PDGFRA−/−^ with PDGF-AA. Compared with parental cells, loss of PDGFRA could not diminish PDGF-AA-induced phosphorylation of EPHA2 (Fig. 2d). In addition, functional inhibition of PDGFRA by IMA did not affect the PDGF-AA-induced phosphorylation of EPHA2, either (Fig. 2e). Using U251 cells, we found that the phosphorylation of EPHA2 did not relay on PDGFRA protein or activation (Supplementary Fig. S2d and S2e). Then, we used inhibitor of EPHA2 (ALW-II-41-27) to pretreat LN18 and U251 cells followed by PDGF-AA stimulation. Intriguingly, ALW-II-41-27 resulted in significant decrease of PDGF-AA-induced activation of AKT, but AXL inhibitor TP0903, which we used as negative control, did not alter the phosphorylation trend of AKT (Supplementary Fig. S2f). We further investigated the PDGF-AA-induced spatiotemporal distribution of EPHA2 in GBM cells by immunofluorescence. Since endosomes are key location for endocytotic RTKs,^33–37^ we used EEA1, a marker of endosome, as a beacon for EPHA2 cellular localization. The results showed that, without PDGF-AA, EPHA2 distributed evenly in cells, and colocalization of EPHA2 and EEA1 were hardly detected (Fig. 2f). At 15 and 30 min following ligand stimulation, most of EPHA2 colocalized with EEA1, and mainly concentrated around the nuclei, implying trafficking towards endosome (Fig. 2f). At 60 min, however, EPHA2 restored distribution as inactivation form and significantly decreased in accordance with western blotting result (Fig. 2f). Together, these results indicated that EPHA2 could be activated by PDGFA in a PDGFRA-independent manner and involved in PDGFA signaling.

Since PDGFA activated EPHA2 in our study, we asked whether PDGFA could interact with EPHA2. Co-IP-MS analysis using LN18^PDGFRA−/−^ showed EPHA2 could interact with PDGFA (Dataset 3), which was confirmed by Co-IP and western blotting in GBM cells (Fig. 2g) or through in vitro protein binding assay (Supplementary Fig. S2g). We then simulated the interaction of PDGF-AA and EPHA2, and the result showed that homodimer of mature PDGFA could interact with EPHA2 extracellular domain (Fig. 2h and Supplementary Fig. S2h). To examine the interaction between EPHA2 and PDGFA, we determined the binding thermodynamics of two peptides using Microcal iTC200. The data showed that recombinant human PDGFRA extracellular domain fused with IgG Fc (rhRA-ECD-Fc), a positive control, could interact with PDGF-AA with ΔH = −1066 ± 116.7 (Supplementary Fig. S2i), indicating spontaneous reaction. Similarly, EPHA2 extracellular domain tagged with histidine (rhA2-ECD-His) and PDGF-AA also produced negative ΔH (−262.3) (Fig. 2i), supporting their interaction. Proximity ligation assay (PLA), which detects in situ direct interactions between two proteins that are less than 40 nm apart,^38,39^ clearly indicated the interaction of PDGF-AA with EPHA2 but the interaction could be significantly reduced by rhRA-ECD-Fc treatment (Fig. 2j and Supplementary Fig. S2j). Thus, EPHA2 might function as a receptor for PDGF-AA. Then we asked whether PDGFRA and EPHA2 could function as a complex together. Co-expression of PDGFRA-Flag with PDGFRA-HA, EPHA2-Flag with EPHA2-GFP, or PDGFRA-HA with EPHA2-Flag showed that PDGFRA and PDGFRA, EPHA2 and EPHA2, or PDGFRA and EPHA2 could form complex in GBM cells (Supplementary Fig. S2k). Indeed, the activation of PDGF-AA-induced PDGFRA phosphorylation could be enhanced by overexpression of EPHA2 but reduced by knockdown of EPHA2 in LN18 cells (Fig. 2K and Supplementary Fig. S2l). With treatment of PDGF-AA, p-AKT(S473) was clearly detected in control LN18 cells but not in LN18 cells with sgPDGFRA and shEPHA2 (Supplementary Fig. S2m). Furthermore, in control LN18 cells, we could detect a temporal increase of PDGF-AA uptake but failed to do so in N18 cells with sgPDGFRA and shEPHA2 (Supplementary Fig. S2n). These data highlighted that EPHA2 might coordinate with PDGFRA to mediate PDGF-AA activity in GBM cells. Together, EPHA2 might function as a novel receptor for PDGFA and mediate PDGFA signaling solely or together with PDGFRA.

### Expression of EPHA2 was correlated with that of PDGF signaling targets in GBM

**Fig. 3:**
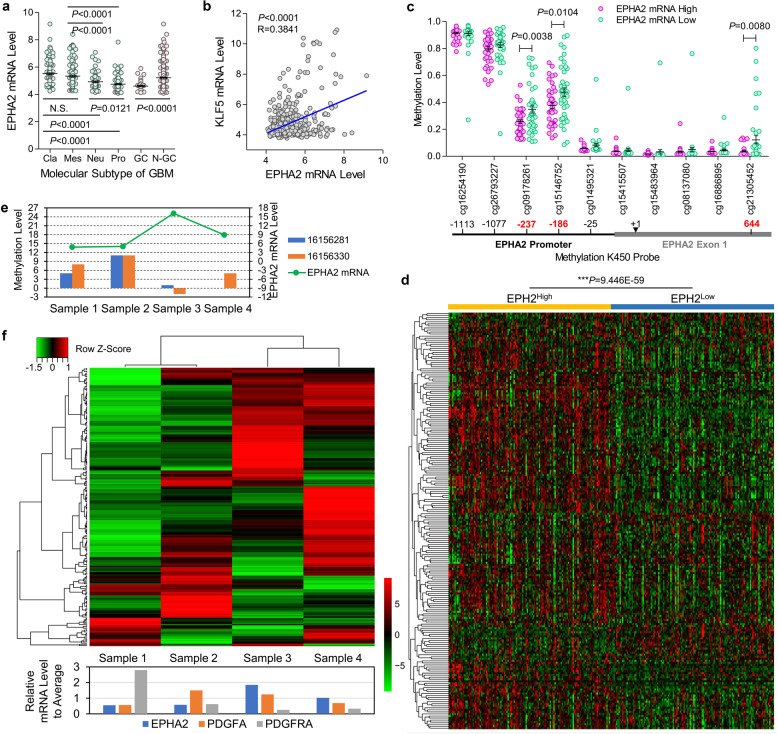
EPHA2 expression regulation and relationship with PDGF downstream targets. **a** EPHA2 expression in different molecular subtypes of GBM in TCGA_GBM database. **b** Pearson correlation of EPHA2 and KLF5 in TCGA_GBM database. **c** Methylation levels of EPHA2 promoter region measured with methylation K450 probes. **d** Heatmap cluster analysis of PDGF signaling target genes with EPHA2High vs. EPHA2Low in TCGA_GBM database. **e** The methylation level in promoter region corresponding to two critical probes at EPHA2 promoter from TCGA_GBM and the EPHA2 mRNA level in four tumor foci from a multifocal GBM patient. **f** Upper: Heatmap cluster analysis of PDGF signaling target genes in the four tumor foci; Lower: mRNA expression of EPHA2, PDGFA, and PDGFRA in the four tumor foci.

Since the involvement of EPHA2 in PDGFA signaling, we examined whether EPHA2 had roles in tumor growth and invasion of GBM cells through MTT assay an Matrigel-coated transwell assay in the context of PDGF-AA stimulation, which indicated that EPHA2 knockdown decreased viability (Supplementary Fig. S3a) and invasiveness (Supplementary Fig. S3b). Then, we investigated the potential expression regulation and downstream targets in GBM. In TCGA_GBM database, we observed that EPHA2 expression in classical and mesenchymal GBM was significantly higher than that in neural and proneural GBM (Fig. 3a). We also evaluated the factors involved in EPHA2 expression regulation. Via gliovis.bioinfo.cnio.es website, we profiled all genes significantly correlated with EPHA2 (Dataset 4). Through combining these genes with the transcription factor list from Uniport (Dataset 5), we observed that KLF5 was ranked No.1 in EPHA2-related transcription factors (Fig. 3b and Supplementary Table S2). It has been known that gene transcription is critically regulated by CpG methylation, which promoted us to explore CpG methylation of EPHA2 gene promoter. According to the methylation K450 probe, we noticed that low methylation at 3 of 10 probes in EPHA2 promoter region were corresponding to high expression of EPHA2 mRNA (Fig. 3c and Supplementary Table S3). Consistently, the three probes showed low methylation levels in classical and mesenchymal subtype GBM but high methylation levels in neural and proneural subtype GBM (Supplementary Fig. S3c). To examine whether EPHA2 expression was involved in PDGF signaling, we defined geneset of PDGF downstream targets through combining several well-described PDGF signaling genesets (Dataset 6) followed by heatmap cluster analysis according to EPHA expression in TCGA_GBM database. Interestingly, PDGF target genes were significantly higher in EPHA2^High^ cases than EPHA2^Low^ cases (*P* = 9.446E−59) (Fig. 3d).

To test the conclusion from TCGA_GBM, we collected four tumor foci from a multifocal GBM patient with four separate lesions at diagnosis^40^ (Supplementary Fig. S3d). Whole-genome sequencing confirmed the four tumor foci harbored typical genetic features of GBM (Supplementary Fig. S3e), including gain and loss of chromosome 7 and 10, respectively, and loss of key tumor suppressors PTEN and CDKN2A/2B.^18,21^ In addition, we observed the amplification of EGFR and PDGFA in all tumor foci and amplification of PDGFRA gene in tumor sample 2 and 3. We then performed RNA sequencing to profile transcriptome of the four tumor foci (Dataset 7) and noticed that KLF5 was also the top 1 transcription factor correlated with EPHA2 in the four samples (Supplementary Fig. S3f and Table S4). We performed whole-genome bisulfite sequencing (Dataset 8) and confirmed that the methylation level in promoter region corresponding to the two critical probes at EPHA2 promoter from TCGA_GBM was negatively related with EPHA2 mRNA level in the four tumor samples (Fig. 3e). Using the transcriptome from the four samples, we performed heatmap cluster analysis on PDGF target genes. Similar to TCGA_GBM database, EPHA2 was correlated with the expression of PDGF target genes. Moreover, PDGFRA expression seemed not so tightly related to PDGF target genes as EPHA2 (Fig. 3f). Together, these data revealed KLF5 and two methylation sites in EPHA2 promoter region as critical regulation elements for EPHA2 expression and confirmed the tight involvement of EPAH2 in PDGF signaling pathway.

### High expression of PDGFRA and EPHA2 enriched oncogenic genesets in GBM cells

**Fig. 4:**
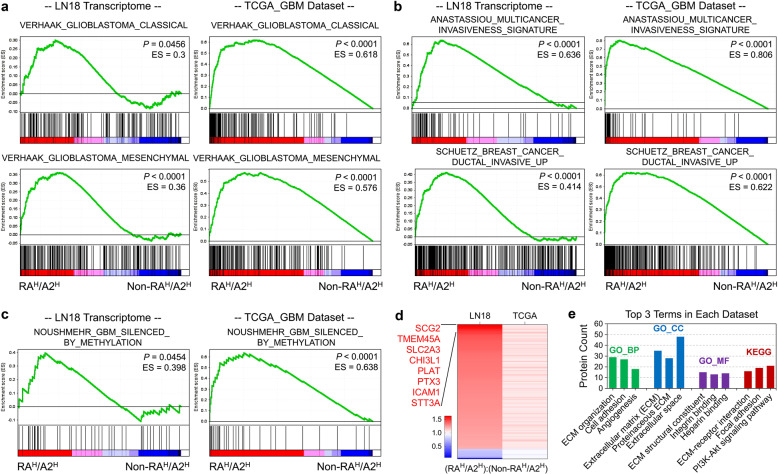
Transcriptomic analyses on PDGFRA and EPHA2 co-upregulation in GBM cells. **a** Enrichment of signature genes of GBM molecular subtype for LN18 cells with co-transfection of EPHA2 and PDGFRA (RA^H^/A2^H^) *vs*. individual transfection (Non-RA^H^/A2^H^) (left two panels), as well as, cases with PDGFRA^High^/EPHA2^High^ (RA^H^/A2^H^) vs. all other (Non-RA^H^/A2^H^) cases from TCGA_GBM mRNA expression dataset (right two panels). **b** Enrichment of signature genes of invasive growth for LN18 cells with RA^H^/A2^H^ *vs*. Non-RA^H^/A2^H^ cases (left two panels), as well as, cases with RA^H^/A2^H^ vs. Non-RA^H^/A2^H^ from TCGA_GBM mRNA expression dataset (right two panels). **c** Enrichment of signature genes of GBM G-CIMP subtype for LN18 cells with RA^H^/A2^H^ vs. Non-RA^H^/A2^H^ (left panel), as well as, cases with RA^H^/A2^H^ vs. Non-RA^H^/A2^H^ cases from TCGA_GBM mRNA expression dataset (right panel). **d** Heatmap graph of consistently altered genes in LN18 cells with RA^H^/A2^H^ vs. Non-RA^H^/A2^H^ (left lane) and cases with RA^H^/A2^H^ vs. Non-RA^H^/A2^H^ cases from TCGA_GBM mRNA expression dataset (right lane). **e** David analysis on consistently altered genes in LN18 cells with RA^H^/A2^H^ vs. Non-RA^H^/A2^H^ (left lane) and cases with RA^H^/A2^H^ vs. Non-RA^H^/A2^H^ cases from TCGA_GBM mRNA expression dataset (right lane). Categories with tops 3 protein counts are showed in the graph.

To understand the overall effects of co-upregulation of PDGFRA and EPHA2 on GBM cells, we profiled transcriptomes of LN18 cells with forced expression of EGFP as control, PDGFRA only, EPHA2 only, or PDGFRA with EPHA2, respectively (Dataset 9). Compared with either PDGFRA or EPHA2 individual expression, co-upregulation of the two genes could significantly enrich mesenchymal and classical signature genes (Fig. 4a), but failed to enrich Proneural and Neural signature genes (Supplementary Fig. S4a). Similar conclusion was drawn from analysis on TCGA_GBM database (Fig. 4a and Supplementary Fig. S4a). We noticed that PDGFRA^High^/EPHA2^High^ in LN18 cells enriched genesets of ANASTASSIOU MULTICANCER INVASIVENESS SIGNATURE and SCHUETZ BREAST CANCER DUCTAL INVASIVE UP (Fig. 4b), which was observed in TCGA_GBM database (Fig. 4b). Moreover, analysis on TCGA_GBM indicated that PDGFRA^High^/EPHA2^High^ enriched hallmark genesets of EPITHELIAL MESENCHYMAL TRANSITION, ANGIOGENESIS, HARRIS BRAIN CANCER PROGENITORS, and CORDENONSI YAP CONSERVED SIGNATURE (Supplementary Fig. S4b), further supporting the oncogenic roles of PDGFRA and EPHA2. G-CIMP subtype of GBM shows low expression of some oncogenes due to DNA methylation-related gene silence and assumes better prognosis than Non-G-CIMP subtype of GBM.^18,41^ GSEA indicated that PDGFRA^High^/EPHA2^High^ transcriptome significantly enriched genes silenced in G-CIMP GBM compared with that of Non-PDGFRA^High^/EPHA2^High^ in both LN18 cells and TCGA_GBM database (Fig. 4c).

We specifically analyzed consistently altered genes in both LN18 cells and TCGA_GBM database in the context of PDGFRA^High^/EPHA2^High^ vs. Non-PDGFRA^High^/EPHA2^High^. 150 genes were significantly altered in both datasets (*P* < 0.01) with 139 upregulated and 11 downregulated (Fig. 4d and Supplementary Table S5). Eight genes (SCG2, TMEM45A, SLC2A3, CHI3L1, PLAT, PTX3, ICAM1, STT3A) were upregulated more than 1.5-fold. Next, we analyzed the relationships of eight significantly upregulated genes with expression of EPHA2, PDGFRA, and PDGFA in TCGA_GBMLGG database via GEPIA 2 (http://gepia2.cancer-pku.cn). The result showed that each of eight genes were positively correlated with combined expression of EPHA2, PDGFRA, and PDGFA (supplementary Fig. S4c). Forced expression of PDGFRA and EPHA2 together in LN18 cells with PDGF-AA treatment could significantly upregulate seven of eight target genes with exception of CHI3L1 (Supplementary Fig. S4d), confirming the regulation of these genes by EPHA2 and PDGFRA. Analysis on the consistently upregulated proteins via David software showed that cell-extracellular matrix interaction-related categories were top ranked in GO and KEGG categories (Fig. 4e), which well supported the effect of PDGFRA and EPHA2 on invasive growth of GBM cells. In addition, angiogenesis and PI3K-AKT signaling pathway were significantly enriched (Fig. 4e), which were consistent with known functions of PDGFRA and EPHA2. Among the top-ranked 8 genes, SCG2, SLC2A3, CHI3L1, PLAT, PTX3, and ICAM1 were found as markers for poor survival of GBM patients using the TCGA_GBM database (Supplementary Fig. S4e). High expression of TMEM45A or STT3A also showed worse prognosis than low expression despite no statistical significance (data not shown). Thus, these significantly upregulated genes might contribute to increased invasive growth of GBM cells induced by PDGFRA and EPHA2 co-expression.

### PDGFRA and EPHA2 were promising pharmaceutical targets for GBM

**Fig. 5:**
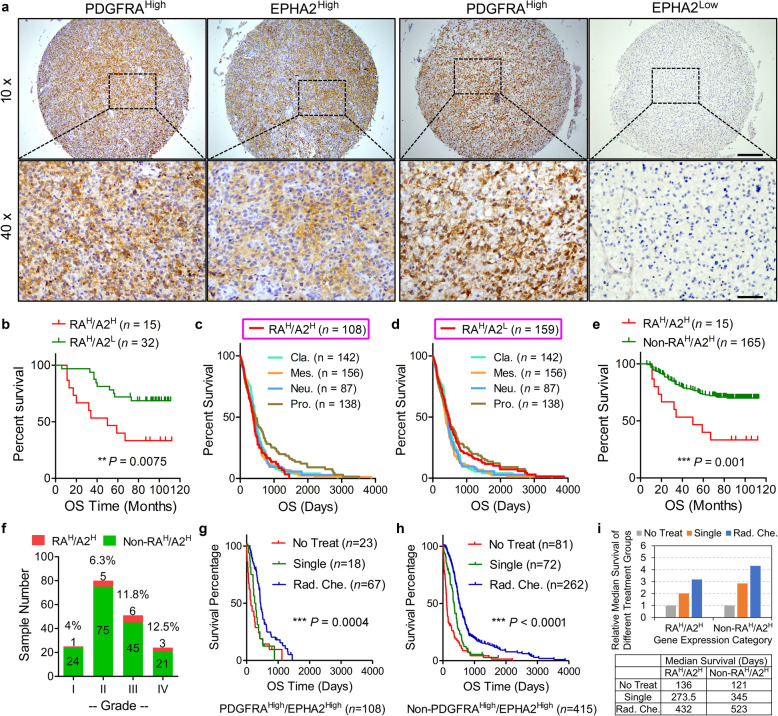
Clinical significance of EPHA2 and PDGFRA in GBM. **a** Representative immunohistochemistry images of EPHA2 and PDGFRA proteins on continuous tissue sections. Scale Bar = 200 μm (upper) and 50 μm (lower). **b** Kaplan–Meier survival analysis on cases with PDGFRA^High^/EPHA2^High^ vs. PDGFRA^High^/EPHA2^Low^ from our glioma cohort. **c** Survival curve comparison between cases with PDGFRA^High^/EPHA2^High^ and different molecular subtypes according to TCGA_GBM mRNA expression dataset. **d** Survival curve comparison between cases with PDGFRA^High^/EPHA2^Low^ and different molecular subtypes according to TCGA_GBM mRNA expression dataset. **e** Kaplan–Meier survival analysis on cases with PDGFRA^High^/EPHA2^High^ vs. all other cases from our glioma cohort. **f** Case count with different protein expression patterns from our glioma cohort according to tumor grades. **g**, **h** Kaplan–Meier survival analysis of different treatment ways under specific gene expression patterns according to TCGA_GBM database. **i** Therapeutic effects of treatment ways on survival time of patients with specific gene expression patterns.

To evaluate protein expression of PDGFRA and EPHA2 using clinical samples of glioma, we collected a 180-case glioma cohort (Cohort-180). Kaplan–Meier survival analysis on Cohort-180 showed that patients with higher-grade gliomas had significantly shortened survival time compare to those with lower-grade gliomas (supplementary Fig. S5a), confirming the reliability of the cohort. We stained the Cohort-180 with antibodies of anti-PDGFRA and anti-EPHA2 antibodies with definition of high expression and low expression according to staining percentage and intensity (supplementary Fig. S5b). Cases with PDGFRA^High^/EPHA2^Low^ showed dramatically improved prognosis compared to those with PDGFRA^High^/EPHA2^High^ (Fig. 5a, b), which was confirmed by TCGA_GBM database (supplementary Fig. S5c). It has been reported that Proneural subtype showed better survival in comparison with the other three subtypes in TCGA_GBM dataset.^15^ Interestingly, the survival curve of PDGFRA^High^/EPHA2^High^ similar to those of Mesenchymal, Classical, and Neural (Fig. 5c), but the survival curve of PDGFRA^High^/EPHA2^Low^ matched with that of Proneural (Fig. 5d), which implied that both proteins might be used as pathological markers to predict prognosis without testing the subtypes of patients. To simplify the potential diagnostic application of the two proteins, we compared PDGFRA^High^/EPHA2^High^ cases with all non-PDGFRA^High^/EPHA2^High^ cases. In Cohort-180, cases with PDGFRA^High^/EPHA2^High^ had worse prognosis than those with non-PDGFRA^High^/EPHA2^High^ (Fig. 5e). PDGFRA^High^/EPHA2^High^ showed increased percentage with glioma progression: 4% (1 in 25 cases) for grade I glioma, 6.3% (5 in 80 cases) for grade II glioma, 11.8% (6 in 51 cases) for grade III glioma, and 12.5% (3 in 24 cases) for GBM (Fig. 5f). In TCGA_GBM database, the percentage of cases with PDGFRA^High^/EPHA2^High^ in Classical (30.3%) and Mesenchymal (24.1%) was much higher than Proneural (12.9%) and Neural (11.5%) (Supplementary Fig. S5d). We then analyzed TCGA_GBMLGG database on the expression of PDGFRA and EPHA2. The data consistently showed that patients with PDGFRA^High^/EPHA2^High^ had shorter survival time than those with PDGFRA^High^/EPHA2^Low^ in all grades and HGGs with statistical significance (*P* < 0.0001 and *P* = 0.0006) and in low-grade glioma without statistical significance (*P* = 0.1179) (supplementary Fig. S5e). In addition, the number of cases with PDGFRA^High^/EPHA2^High^ in HGG was much higher than that in low-grade glioma (90 vs. 38), but the number of cases with PDGFRA^High^/EPHA2^Low^ in HGG was lower than that in low grade glioma (101 vs. 121) (Supplementary Fig. S5f). We also performed IHC staining using anti-PDGFA and anti-phospho-AKT(S473) antibodies (Supplementary Fig. S5g). The *χ*^2^ test indicated that high expression of PDGFA was positively related with high levels of p-AKT(S473) (Supplementary Fig. S5h). It was noted most of PDGFA^High^/p-AKT(S473)^High^ cases (74 in 78) had high expression of PDGFRA and EPHA2, either individually or together (Supplementary Fig. S5h). In addition, correlation between PDGFA and p-AKT(S473) was much more significant in cases with high expression of PDGFRA and/or EPHA2 than those with low expression of PDGFRA and EPHA2 (*P* = 2.7e−8 vs. *P* = 0.018) (Supplementary Fig. S5h). Thus, the distribution feature of PDGFRA and EPHA2 implied that the two proteins were related with malignant phenotype of glioma.

We further evaluated the significance of PDGFRA and EPHA2 expression for treatment effects. The result showed that single regimen treatment (radiation or chemotherapy) did not show benefit on all patients, and radio-chemo therapy mildly prolonged patient survival with PDGFRA^High^/EPHA2^High^ (Fig. 5g). However, both single regimen and radio-chemo therapy significantly improved survival of patients with non-PDGFRA^High^/EPHA2^High^ (Fig. 5h). In addition, clinical therapy prolonged median survival time in patients with non-PDGFRA^High^/EPHA2^High^ much more than those with PDGFRA^High^/EPHA2^High^ (224 days vs. 137.5 days for single regimen and 402 days vs. 296 days for radio-chemo therapy) (Fig. 5i). Therefore, our clinical analysis confirmed that concurrent expression of PDGFRA and EPHA2 could be promising prognostic markers and therapeutic targets.

### Simultaneously targeting EPHA2 and PDGFRA suppressed growth of GBM cells in vitro and in vivo

**Fig. 6:**
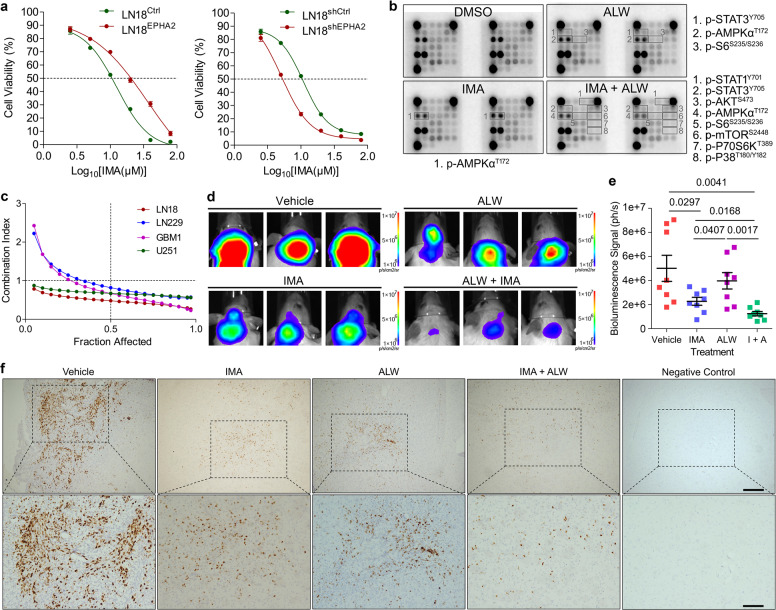
Co-inhibition of PDGFRA and EPHA2 synergetically inhibits GBM cells. **a** IC_50_ measurement of LN18 cells with forced expression of EPHA2 (left panel) or knockdown of EPHA2 (right panel) through MTT assay. **b** Antibody array analysis of LN18 cells treated with vehicle, EPHA2 inhibitor (ALW), and PDGFRA inhibitor (IMA). Significant changed proteins are labeled with frame and listed separately. **c** MTT assay-based drug combination evaluation in four GBM cell lines. **d** Representative images of orthotopic growth of U251 cells treated with vehicle, IMA, ALW, or IMA + ALW. **e** Statistic graph of tumor size using bioluminescence signal intensity. *n* = 8 for each group. **f** Representative immunohistochemistry images of Ki67 on tissue sections from mice with orthotopic GBM tumors treated by vehicle, IMA, ALW, or IMA + ALW. Scale Bar = 200 μm (upper) and 100 μm (lower).

Since PDGFA could activated EPHA2 bypassing PDGFRA, we speculated that GBM cells with high EPHA2 might be resistant to IMA but GBM cells with low EPHA2 might be sensitive to IMA. As expected, we found that IC_50_ of LN18^EPHA2^ to IMA was higher than that of LN18^Ctrl^. Consistently, IC_50_ of LN18^shEPHA2^ to IMA was lower than that of LN18^shCtrl^ (Fig. 6a). Similarly, IC_50_ of U251^EPHA2^ to IMA was higher than that of U251^Ctrl^ and IC_50_ of U251^shEPHA2^ to IMA was lower than that of U251^shCtrl^ (Supplementary Fig. S6a). Growth curve measurement through MTT indicated that the decreased expression of EPHA2 sensitized GBM cells to IMA (Supplementary Fig. S6b). GSEA on TCGA_GBM database consistently showed that high expression of EPHA2 significantly enriched genes upregulated in IMA resistant patients (GSE155800) and cells ([MAHADEVAN GIST MORPHOLOGICAL SWITCH]) (Supplementary Fig. S6c). To evaluate whether there was coordination of PDGFRA and EPHA2 upon PDGF-AA stimulation, we used IMA and ALW alone or in combination to treat GBM cells. Antibody array result showed that the combination of two inhibitors exerted significantly stronger inhibition on the activation of PDGFA downstream targets than each inhibitor alone (Fig. 6b). Furthermore, MTT assay confirmed that the two inhibitors showed synergetic effects in several GBM cell lines (Fig. 6c). Colony formation assay consistently proved that the combination of IMA and ALW more potently suppressed expansion of GBM cells than either one of the two inhibitors (Supplementary Fig. S6d). Thus, our results revealed that EPHA2 might coordinate with PDGFRA to augment PDGF-AA effects in GBM cells.

We then examined the inhibitory effects of the two inhibitors using orthotopic mouse model. The SCID mice was orthotopically inoculated with U251 cells together with PDGFA virus, which could effectively promote in vivo growth of GBM cells in brain (Supplementary Fig. S6e and S6f). One week later, the mice were treated with PBS, IMA (25 mg/kg), ALW (10 mg/kg), or IMA + ALW via intraperitoneal injection. The data showed that IMA or ALW alone could reduce the size of GBM but the effects was not statistically significant (*P* > 0.05). Combination of the two inhibitors, however, significantly inhibited the tumor growth compared with PBS or ALW treatment (Fig. 6d, e) and the inhibitory effects were supported by Ki67 staining (Fig. 6f). Parallel animal experiments for survival analysis suggested that combination of ALW and IMA could improve survival of mice with GBM (Supplementary Fig. S6g). IMA (STI571, CGP-57148B) is known as a selective blood-brain barrier-permeable PDGFR antagonist.^42,43^ We stained p-EPHA2 in xenograft with or without ALW, and the data revealed marked decrease of p-EPHA2 with treatment of ALW (supplementary Fig. S6h), implying the permeability of ALW towards BBB. Thus, the tumor suppression was resulted from inhibitors and simultaneously targeting PDGFRA and EPHA2 could effectively repress GBM growth in vivo.

### PDGFA, but not EFNA1, endowed oncogenic roles of EPHA2 in GBM cells

**Fig. 7:**
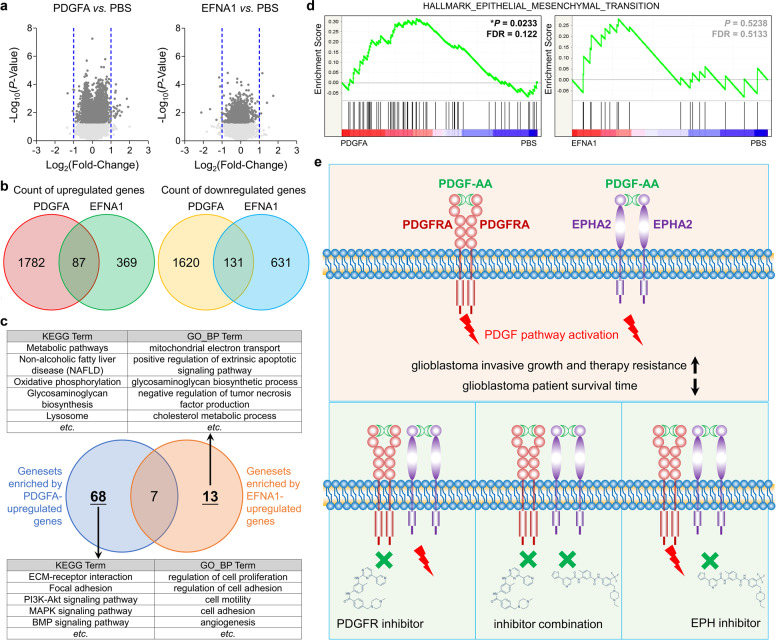
Analysis on transcriptomes of LN18^PDGFRA−/−^ treated with PBS, PDGFA or EFNA1. **a** Volcano graph of genes affected by PDGFA or EFNA1 vs. PBS. **b** Venn diagram of genes affected by PDGFA or EFNA1 vs. PBS. **c** Venn diagram of genesets enriched by PDGFA or EFNA1-upreguleted genes. **d** GSEA graph of EMT hallmark signature enriched by PDGFA or EFNA1-regulated genes. **e** Schematic diagram of EPHA2 and PDGFRA-mediated PDGFA function in GBM cells. Both PDGFRA and EPHA2 mediate PDGFA function to promote invasive growth and therapeutic resistance of GBM cells (upper panel). Single pharmaceutical inhibition of EPHA2 or PDGFRA cannot effectively suppress PDGFA activity due to the existence of compensate pathway. Concurrent inhibition of PDGFRA and EPHA2, however, potently block PDGFA signaling transduction (lower panels).

It has been known that EFNA1 is a major cognate ligand of EPHA2 in vivo, but the function of EFNA1 is thought to impair EPHA2 activity in glioma cells,^44–46^ which promoted us to investigate the difference of PDGFA/EPHA2 axis and EFNA1/EPHA2 axis. To avoid the interference of PDGFRA on PDGFA-EPHA2 axis, we treated PDGFRA^−/−^ GBM cells with PBS as control, recombinant soluble human EFNA1 with Fc-tag, and recombinant human PDGFA homodimer followed by RNA sequencing to profile transcriptomes (Dataset 10). The RNA sequencing data showed that genes altered by PDGFA and EFNA1 were few overlayed (Fig. 7a, b). David analysis showed that genesets enriched by PDGFA-upregulated genes (Cutoff: *P* < 0.05 and Fold Chang > 1.25) were obviously distinguished from those enriched by EFNA1-upregulated genes (Cutoff: *P* < 0.05 and Fold Chang > 1.25) (Fig. 7c and Supplementary Table S6). The former included cell mobility and PDGF-related genesets, but the latter included metabolism, apoptosis, and lysosome-related genesets. In addition, PDGFA could enrich EMT geneset, but EFNA1 failed to do so (Fig. 7d). Thus, EFNA1 could not induce EPHA2 oncogenic roles in GBM, which was consistent with previous reports.^44–48^

## Discussion

It has been documented that PDGFA/PDGFRA is highly expressed in GBM and plays critical roles in gliomagenesis, but targeting PDGFRA by small molecule inhibitors do not show therapeutic significance in clinic. In this study, we found that, besides PDGFRA, EPHA2 could also mediate PDGFA signaling pathway in a PDGFRA-independent manner. In addition, EPHA2 and PDGFRA could also function together to enhance PDGFA functions. Therefore, inhibition of PDGFRA or EPHA2 only was not sufficient to block PDGFA function but concurrent suppression of the two kinases could be promising regimen for GBM treatment (Fig. 7e).

EPAH2 belongs to the largest RTK subfamily—EPH receptor family, and is the most frequently altered EPH members in cancers.^49^ In GBM, EPHA2 functions as a mitogen^50^ and high expression of EPHA2 is correlated with poor survival of patients.^45,46^ Our data clearly showed that EPHA2 was required for viability and invasiveness of GBM cells and significantly upregulated genes by EPHA2 were also involved in malignant phenotype of GBM. Interestingly, oncogenic roles of EPHA2 in GBM are independent of its cognate ligand —type-A ephrin,^45,46^ implying other unidentified proteins may act as ligands of EPHA2. In this work, we found that PDGFA interacted with and activated EPHA2 in GBM cells, and furthermore, in vitro assay and three-dimension structure simulation indicated PDGFA as a EPHA2 ligand. The identification of PDGFA as a ligand of EPHA2 might explain the functional regulation of EPHA2 in GBM. Moreover, EPHA2 has been known to play important roles in glioblastoma stem cells or stem-like cells.^46,51–53^ We also observed that higher protein level of EPHA2 in GBM cells cultured in neurosphere medium than in attachment medium (Data not shown). Thus, targeting EPHA2 not only reduced glioblastoma stemness but also suppressed PDGF-AA-induced tumor growth.

Although four PDGF ligands have been identified, the frequency of genetic alteration (gain of copy number) and mRNA increase of PDGFA was significantly higher than those of PDGFB, C, and D in GBM, indicating the critical involvement of PDGFA in GBM. Interestingly, we did not detect interaction between EPHA2 with PDGFB, PDGFC, or PDGFD (Data not shown), implying that interaction between EPHA2 with PDGFA might be specific in GBM cells. The only identified receptor of PDGFA is PDGFRA, which is one of the most typical features of Proneural GBM, and interestingly, all subtypes are thought to evolve from proneural-like glioma precursor and most secondary GBMs highly resemble proneural phenotype.^18^ PDGFRA functions as a putative driver gene during glioma development induced by intracranial radiation.^19,20^ These findings reveal PDGFA/PDGFRA as a potential therapeutic target, but targeting the signaling axis has failed in clinical trials. Since EPHA2 could be activated by PDGFA even without PDGFRA activation (IMA treatment) or PDGFRA expression (PDGFRA^−/−^), the cells with co-upregulation of PDGFRA and EPHA2 might be insensitive to PDGFRA inhibitor alone, but could be sensitive to combination of PDGFRA inhibitor and EPHA2 inhibitor. Thus, our current results provided an explanation for the failed clinical trials targeting PDGFRA.

In the study, we used four tumor foci from a multifocal GBM patient to examine the regulation and function of EPHA2. About 10–20% of GBM patients are diagnosed with more than one tumor lesion or multifocal GBM.^1,54^ Patients with multifocal GBM have shortened overall survival compared to those with one GBM mass or unifocal GBM and are resistant to current therapeutic measures.^55,56^ Various tumor foci from same multifocal GBM patient are actually evolved from monoclonal origin.^57^ Therefore, analysis on tumor foci of same multifocal GBM patient could accurately profile evolution difference among these tumor foci without interference of individual genetic and epigenetic difference. Using this model together with TCGA_GBM database, we revealed transcription factor KLF5 and two methylation sites in EPHA2 promoter region as potential regulation elements of EPHA2 transcription, which needed further pursue in following work. Moreover, our findings suggested that EPHA2 high expression was correlated with high expression of PDGF signaling targets, confirming the tight involvement of EPHA2 in PDGFA function.

In combination with TCGA_GBM database, we noticed that co-expression of PDGFRA and EPHA2 significantly enriched genesets of mesenchymal and classical but not proneural and neural, and moreover, several invasive growth-related genesets were also enriched by concurrent expression of the two proteins. we collected a panel of potential target genes upregulated by EPHA2 and PDGFRA and found that the functions of these genes were mainly in the extracellular matrix, cell adhesion, angiogenesis, and PI3K-AKT, which were tightly correlated with malignant phenotypes of GBM with high expression of PDGFRA and EPHA2. Further analysis on clinical treatment efficacy using TCGA_GBM database showed that GBM with PDGFRA and EPHA2 was insensitive to radiation or/and chemotherapy but GBM without PDGFRA and EPHA2 responded well to clinical treatment strategy. Therefore, pathological examination of PDGFRA and EPHA2 might be valuable for the prediction of survival and clinical treatment efficacy.

In addition, our study for the first time profiled the regulation network of PDGFA-associated proteins, including kinases, protein modification regulators, and protein trafficking regulators. It is well-known that growth factor-induced activation of RTKs, including EGFR, FGFR, IGF1R, PDGFRA, and PDGFRB, are dependent on receptor internalization, trafficking, and endocytosis.^33–37^ Extensive researches on EGFR endocytosis through high-resolution proteomics depict endocytosis-related temporal interactomes of EGFR, which form a dynamic regulation network for EGFR activation.^33–35^ From the EGF-induced EGFR interactomes, several novel EGFR regulators have been identified as potential therapeutic targets for cancers driven by EGFR,^33–35^ and hence blocking the interaction between RTK with its regulators represents a novel strategy to target the overactivated RTK.^58^

Altogether, our study for the first time profiled the interactome of PDGFA in GBM cells and revealed a critical interaction between PDGFA and EPHA2, which provided new insights on PDGFA/PDGFRA and PDGFA/EPHA2 signaling axes in GBM. Moreover, our work implied that EPHA2 and PDGFRA might be therapeutic targets for GBM with high expression of both proteins, emphasizing that the molecular mechanisms underlying PDGFA signaling activation by new binding partners need to be clarified in detail for application of PDGFA-related therapeutic strategies on GBM treatment.

## Materials and methods

### GBM samples

Glioma tissue microarrays (HBraG181Su01 and HBraG169Su01) were purchased from Shanghai Outdo Biotech CO., LTD. (http://www.superchip.com.cn/index.html). This study was approved by the Medical Ethical Committees of Southwest Hospital, Third Military Medical University. A 47 years old male patient with multifocal GBM patient was hospitalized in the Department of Neurosurgery, Xinqiao Hospital of Third Military Medical University in 2019 and subjected to neuro-navigation and fluorescein-guided surgery after a clear evaluation 3 days later. Four separate tumor foci were removed with sample A in left Frontal Lobe, sample B in left Frontal Lobe, sample C in left Gyrus Cinguli, and sample D in left Parietal Lobe. The patient was dead after 11 months after surgery.^40^ This study was approved by the Medical Ethical Committees of Xinqiao Hospital and Southwest Hospital, Third Military Medical University. Written informed consents were obtained from the patients.

### Intracranial GBM xenografts and treatment

All animal experiments were approved by the Institutional Animal Care and Use Committee of the Southwest Hospital in accordance with the Guide for the Care and Use of Laboratory Animals. In brief, 5 × 10^5^ U251 GBM cells expressing luciferase reporter together with lentivirus control or lentivirus expressing PDGFA were suspended in 10 μl of PBS and transplanted into the right frontal lobe of 6-week-old NOD/SCID mice from Laboratory Animal Centre at Southwest Hospital. To examine the combined effects of Imatinib (IMA) and ALW-II-41-27 (ALW) treatment, mice were treated with vehicle control (i.p.), IMA (25 mg/kg, i.p., Selleckchem, S1026), ALW (10 mg/kg, i.p., MedChemExpress, HY-18007), or the combination of IMA and ALW. Mice bearing xenografts were given 8 cycles of treatment since Day 7 after tumor implantation. Tumor growth was monitored by bioluminescence imaging using In Vivo Imaging System (IVIS) Spectrum (Guangzhou Biolight Biotechnology). At the end of experiment, mouse brains were collected and subjected to formalin fixation and tissue section for immunohistochemistry.

## Supplementary information

SUPPLEMENTAL MATERIAL

Dataset 1 - 10

## Data Availability

All data are available in the manuscript and its supplemental materials.
