# Supplementary material for: EPHA2 mediates PDGFA activity and functions together with PDGFRA as prognostic marker and therapeutic target in glioblastoma
Source: Signal Transduct Target Ther. 2022 Feb 2;7:33. doi: 10.1038/s41392-021-00855-2 (PMC8807725; doi:10.1038/s41392-021-00855-2)
Supplement: Supplementary file 1 — SUPPLEMENTAL MATERIAL [file 41392_2021_855_MOESM1_ESM.docx]

Supplementary Materials for

EPHA2 mediates PDGFA activity and functions together with PDGFRA as prognostic marker and therapeutic target in glioblastoma

Qu-Jing Gai^1,^*, Zhen Fu^1^^,^*, Jiang He^1,^*, Min Mao^1^, Xiao-Xue Yao^1^, Yan Qin^1^, Xi Lan^1^, Lin Zhang^1^, Jing-Ya Miao^1^, Yan-Xia Wang^1^, Jing Zhu^1^, Fei-Cheng Yang^1^, Hui-Min Lu^1,2^, Ze-Xuan Yan^1^, Fang-Lin Chen^1,3^, Yu Shi^1^, Yi-Fang Ping^1^, You-Hong Cui^1^, Xia Zhang^1^, Xindong Liu^1^, Xiao-Hong Yao^1^, Sheng-Qing Lv^4,#^, Xiu-Wu Bian^1,#^, and Yan Wang^1,#^

Correspondence to: [wang_yan1977@hotmail.com](mailto:wang_yan1977@hotmail.com); [bianxiuwu@263.net](mailto:bianxiuwu@263.net); [lvsq0518@hotmail.com](mailto:lvsq0518@hotmail.com)

**This PDF file includes:**

Materials and Methods

Figures. S1 to S6

Tables S1 to S6

**Other Supplementary Materials for this manuscript include the following:**

Dataset 1 to 10:

Dataset 1. Gene expression matrix of samples from TCGA_GBM according to "High" and "Low" definition of PDGFA and PDGFRA.

Dataset 2. PDGFA-associated proteins in LN18 cells.

Dataset 3. List of EPHA2-binding proteins in LN18PDGFRA-/- under different treatment conditions with Exp. q-value < 0.01.

Dataset 4. List of genes significantly correlated with EPHA2 in TCGA_GBM database.

Dataset 5. List of all transcription factors from Uniport.

Dataset 6. Well-described PDGF signaling genesets.

Dataset 7. Transcriptomes of four tumor foci and blood control from a multipale GBM patient.

Dataset 8. Methylation read of site 10000000 - 20000000 in chromosome 1 of four tumor foci from a multiple GBM patient.

Dataset 9. FPKM value from RNA sequencing on LN18 cells transfected with PDGFRA, EPHA2, PDGFRA+EPHA2 and GFP as control.

Dataset 10. FPKM value from RNA sequencing on LN18 with PDGFRA knockout cells treated with PBS, recombinant human PDGF-AA, or recombinant soluble human EFNA1.

Materials and Methods

**Plasmids**

PDGFRA CRISPR/Cas9 KO plasmid (h2) (#sc-400107-KO-2), PDGFRA HDR plasmid (h2) (sc-400107-HDR-2), and EPHA2 shRNA plasmid (#sc-29304-SH) were purchased from Santa Cruz Biotechnology (https://www.scbt.com/home). Differently tagged pCMV3-PDGFRA and pCMV3-EPHA2 expression plasmid were purchased from Sino Biological (https://cn.sinobiological.com/) and the full insert reading frame was subcloned into pCDH-CMV-MCS-EF1α-Puro lentivector (#CD510B-1), which was purchased from System Biosciences (<https://systembio.com/>), for stable clone establishment. pCDH-CMV-EGFP-EF1α-Puro was constructed through inserting EGFP coding frame into MCS of pCDH-CMV-MCS-EF1α-Puro. Lentivirus and Lentivirus expressing PDGFA were purchased from Shanghai Sunbio medical technology (Shanghai, China).

**Antibodies and reagents**

Anti-β-actin (#AF0003) was from Beyotime Biotechnology. Anti-PDGFA (#sc-9974) and anti-His tag (#sc-8036) were from Santa Cruz Biotechnology. Anti-Flag (#F1804), Anti-FLAG Magnetic Beads (#M8823), Protein A Magnetic Beads (#GE28-9513-78), Protein G Magnetic Beads (#GE28-9513-79), and 3-(4,5-dimethylthiazol-2-yl)-2,5-diphenyltetrazolium bromide (MTT) (#M5655) were from Sigma-Aldrich. Antibodies targeting PDGFRA (#3174), phospho-PDGFRA (#24188), EPHA2 (#6997), phospho-EPHA2 (#3970), AKT (#4685), phospho-AKT (#4060), HA tag (#3724), HRP-anti-Mouse IgG (#7076), HRP-anti-Rabbit IgG (#7074), Normal Mouse IgG1 (#5415), Normal Rabbit IgG (#2729), EEA1 (#48453), Alexa Fluor® 594-anti-Mouse IgG (#8890), Alexa Fluor® 488-anti-Rabbit IgG (#4412), ProLong® Gold Antifade Reagent with DAPI (#8961), and PathScan® Intracellular Signaling Array Kit (Chemiluminescent Readout) (#7323) were from Cell Signaling Technology. Recombinant Human PDGF-AA (#100-13A) and BB (#100-14B) were from PeproTech. Recombinant Human PDGFRA extracellular domain with human FC tag (#10556-H02H), recombinant Human EPHA2 extracellular domain with His tag (#13926-H08H), and recombinant soluble human EFNA1 with Fc-tag (#10882-H02H) were from Sino Biological. EPHA2 inhibitor - ALW-II-41-27 (#HY-18007) was from MCE. PDGFR inhibitor - Imatinib Mesylate (STI571) (#S1026), AXL inhibitor - Dubermatinib (TP-0903) (#S7846), FYN inhibitor- PP1 (#S7060), TGFBRII inhibitor - LY2109761 (#S2704), and MG-132 (#S2619) were from Selleck.

**Proximity ligation assay (PLA)**

PLA was performed using Duolink system to detect interaction between PDGFA and EPHA2. Duolink In Situ Detection Reagents Green (DUO92014, Sigma-Aldrich), Probe Anti–Rabbit MINUS (DUO92005-100RXN, Sigma-Aldrich) and PLA Probe Anti–Mouse PLUS (DUO92001-100RXN, Sigma-Aldrich) were used in PLA assay according to the manufacturer’s instructions. DAPI were used to stain nuclei.

**Cell culture**

293T cell line and human GBM cell lines LN18, LN229, U251 were obtained from the ATCC ([www.ATCC.org](http://www.ATCC.org)). GBM1 is primary GBM cell line ^1^. All cells maintained at 37°C in a 5% CO_2_ incubator DMEM/F12 plus 10% fetal bovine serum. All cell lines were characterized as mycoplasma negative and validated by STR DNA fingerprinting using the AmpFLSTR Identifiler kit (#4322288, ThermoFisher) according to the manufacturer's instructions semiannually. The STR profiles were compared with known ATCC fingerprints and with the Cell Line Integrated Molecular Authentication database version 0.1.200808 (http://archive.is/http://bioinformatics.istge.it/clima/; Nucleic Acids Research 37: D925-D932, PMCID: PMC2686526). The STR profiles matched known DNA fingerprints or were unique. Primary GBM cell lines were established in our department under the approval of medical ethics committees of Southwest Hospital and Army Medical University ^2^. Tumor sphere of GBM cell lines were maintained in serum-free neural stem cell culture medium, containing DMEM/F12 (Gibco), B27 supplements (Gibco), human recombinant basic FGF (20 ng/ml; Upstate, Lake Placid, NY, USA), and human recombinant EGF (20 ng/ml; Sigma-Aldrich, St Louis, MO, USA). Second or more passaged spheres were used in experiments. Sphere cells were dissociated with Accutase (Millipore, Bedford, MA, USA) during passage.

**Transfection and lentiviral infection**

For glioma cells, all plasmids were transfected using Lipofectamine 3000 (#L3000015, ThermoFisher) according to the manufacturer's instructions. The cells were harvested for mRNA extraction or protein extraction after 48 hours of transfection. For lentiviral infection, vector plasmids and packaging plasmids were co-transfected into 293T cells using Lipofectamine 2000 (#11668019, ThermoFisher), and the lentiviruses were concentrated as described previously ^3^. The generated viruses were used for cell infection in the presence of polybrene (#TR-1003-G, EMD Millipore). After infection, stable cells were isolated by selection for resistance to puromycin or by fluorescence-activated cell sorting.

**Immunohistochemistry**

Glioma tissue microarray with 180 cases (#HBraG180Su01) were purchased from Shanghai Outdo Biotech (<http://www.superchip.com.cn/biology/tissue.html>). The immunohistochemistry (IHC) was performed according to our previous study ^4^. The evaluation of IHC staining was performed as below. Five most characteristic high-power fields (× 400 magnification) per tissue section were manually selected using an Olympus BX51 microscope (Olympus, Tokyo, Japan). The percentage of signal positive tumor cells to all tumor cells were counted and classified as more than 50% (+++), between 25% to 50% (++), between 5% to 25% (+), and less than 5% (-). Then, cases with “+++” and “++” were considered as high expression of target proteins. Otherwise, the cases were defined as low expression of target proteins.

**Immunofluorescence**

The immunofluorescence of glioma cell lines was performed using anti-EPHA2 and anti-EEA1 with DAPI for nuclei staining as previously described ^4^.

**Immunoprecipitation, western blotting, and antibody array blotting**

Immunoprecipitation and immunoblotting were performed as previously described ^5^, and antibody array blotting was performed according to manufacturer’s instruction.

**Mass spectrometry, protein identification and label-free quantification**

In-gel digestion, HPLC, mass spectrometry were performed as previously described ^6^. Proteome Discoverer (PD, V1.4, ThermoFisher) with Mascot (Mascot V2.3, Matrix Science) was used to search raw data against Human RefSeq database (the 2013.07.04). Mass tolerance for precursor ions was set to 20 ppm; mass tolerances of fragment ions were 0.02 and 0.5 Da for Q Exactive and LTQ Orbitrap VelosPro, respectively. Carbamidomethylation of cysteine, oxidation of methionine, acetylation of protein N-terminal were included as variable modifications. A maximum of one missed cleavage was allowed. All assigned peptides were filtered with 1% false discovery rate (FDR) at peptide level. We only kept identifications with >= 2 unique peptides (1% FDR and ion score >20), which was stricter than 1% FDR at the protein level. All identified peptides were quantified with peak areas derived from their MS1 intensity. The process was as followed: 1) MS raw data were converted to the MS-platform independent mzXML format; 2) the spectral assignments from PD1.4 were then channeled through an in-housed pipeline to construct Extracted Ion Chromatogram (XIC) peaks with their corresponding intensity values included in mzXML data. For protein quantification, intensity based absolute quantification (iBAQ) algorithm ^6^ was used. To normalize the differences in loading amounts among samples, we then converted iBAQ value to FOT (fraction of total) - iBAQ value of each protein divided by the sum of all iBAQ values of all proteins in the sample. FOTs of most proteins in a sample were very small and more than five decimal values were common. These small numbers would be visually difficult for human eyes. Therefore, we multiplied the FOT number with 105 to obtain iFOT5 to make easier visualization of values. All missing values were substituted with zero.

**RNA sequencing and data processing**

**(1) Library preparation for Transcriptome sequencing**

Total RNA of cells was extracted using TRIzol reagent (15596026 ThermoFisher, USA) according to manufacturer’s instruction. A total amount of 2 μg RNA per sample was used as input material for the RNA sample preparations. Sequencing libraries were generated using NEBNext® UltraTM RNA Library Prep Kit for Illumina® (NEB, USA) following manufacturer’s recommendations and index codes were added to attribute sequences to each sample. Briefly, mRNA was purified from total RNA using poly-T oligo-attached magnetic beads. Fragmentation was carried out using divalent cations under elevated temperature in NEBNext First Strand Synthesis Reaction Buffer(5X). First strand cDNA was synthesized using random hexamer primer and M-MuLV Reverse Transcriptase (RNase H-). Second strand cDNA synthesis was subsequently performed using DNA Polymerase I and RNase H. Remaining overhangs were converted into blunt ends via exonuclease/polymerase activities. After adenylation of 3’ ends of DNA fragments, NEBNext Adaptor with hairpin loop structure were ligated to prepare for hybridization. In order to select cDNA fragments of preferentially 150~200 bp in length, the library fragments were purified with AMPure XP system (Beckman Coulter, Beverly, USA). Then adaptor-ligated cDNA at 37°C for 15 min followed by 5 min at 95 °C before PCR. Then PCR was performed with Phusion High-Fidelity DNA polymerase, Universal PCR primers and Index (X) Primer. At last, PCR products were purified (AMPure XP system) and library quality was assessed on the Agilent Bioanalyzer 2100 system.

**(2) Clustering and sequencing**

The clustering of the index-coded samples was performed on a cBot Cluster Generation System using TruSeq PE Cluster Kit v3-cBot-HS (Illumia) according to the manufacturer’s instructions. After cluster generation, the library preparations were sequenced on an Illumina Hiseq platform and 125 bp/150 bp paired-end reads were generated.

**(3) Quality control**

Raw data (raw reads) of fastq format were firstly processed through in-house perl scripts. In this step, clean data (clean reads) were obtained by removing reads containing adapter, reads containing ploy-N and low-quality reads from raw data. At the same time, Q20, Q30 and GC content the clean data were calculated. All the downstream analyses were based on the clean data with high quality.

**(4) Reads mapping to the reference genome**

Reference genome and gene model annotation files were downloaded from genome website directly. Index of the reference genome was built using Bowtie v2.2.3 and paired-end clean reads were aligned to the reference genome using Hisat2 v2.0.5. We selected Hisat2 as the mapping tool for that Hisat2 can generate a database of splice sets based on the gene model annotation file and thus a better mapping result than other non-splice mapping tools.

**(5) Quantification of gene expression level**

HTSeq was used to count the reads numbers mapped to each gene. And then FPKM of each gene was calculated based on the length of the gene and reads count mapped to this gene. FPKM, expected number of Fragments Per Kilobase of transcript sequence per Millions base pairs sequenced, considers the effect of sequencing depth and gene length for the reads count at the same time, and is currently the most used method for estimating gene expression levels ^7^.

**(6) Differential expression analysis**

Differential expression analysis was performed using the edgeR package. edgeR is one of the most popular Bioconductor packages for assessing differential expression in RNA-seq data. It is based on the negative binomial (NB) distribution and it models the variation between biological replicates through the NB dispersion parameter. This method is immediately able to handle complex experimental designs. Genes with an adjusted P-value <0.05 found by DESeq were assigned as differentially expressed ^8^. Differential expression analysis of two conditions was performed using the DEGSeq R package. The P values were adjusted using the Benjamini & Hochberg method. Corrected P-value of 0.005 and log2(Fold change) of 1 were set as the threshold for significantly differential expression.

**Whole Genome Sequencing**

**(1) DNA Quantification & Qualification**

The quality of isolated genomic DNA was verified by using these two methods in combination: DNA degradation and contamination were monitored on 1% agarose gels. DNA concentration was measured by Qubit® DNA Assay Kit in Qubit® 2.0 Flurometer (Invitrogen, USA).

**(2) Library Preparation**

A total amount of 0.5 μg DNA per sample was used as input material for the DNA library preparations. Sequencing library was generated using Truseq Nano DNA HT Sample Prep Kit (Illumina USA) following manufacturer’s recommendations and index codes were added to each sample. Briefly, genomic DNA sample was fragmented by sonication to a size of 350 bp. Then DNA fragments were endpolished, A-tailed, and ligated with the full-length adapter for Illumina sequencing, followed by further PCR amplification. After PCR products were purified (AMPure XP system), libraries were analyzed for size distribution by Agilent 2100 Bioanalyzer and quantified by real-time PCR (3nM).

**(3) Clustering & Sequencing**

The clustering of the index-coded samples was performed on a cBot Cluster Generation System using Hiseq X PE Cluster Kit V2.5 (Illumina) according to the manufacturer’s instructions. After cluster generation, the DNA libraries were sequenced on Illumina Hiseq platform and 150 bp paired-end reads were generated.

**(4) Quality Control**

The original fluorescence image files obtained from Hiseq platform are transformed to short reads (Raw data) by base calling and these short reads are recorded in FASTQ format, which contains sequence information and corresponding sequencing quality information. Sequence artifacts, including reads containing adapter contamination, low-quality nucleotides and unrecognizable nucleotide (N), undoubtedly set the barrier for the subsequent reliable bioinformatics analysis. Hence quality control is an essential step and applied to guarantee the meaningful downstream analysis.

The steps of data processing were as follows:

Discard paired reads if either one read contains adapter contamination (>10 nucleotides aligned to the adapter, allowing ≤ 10% mismatches);

Discard paired reads if more than 10% of bases are uncertain in either one read;

Discard paired reads if the proportion of low quality (Phred quality <5) bases is over 50% in either one read.

All the downstream bioinformatics analyses were based on the high-quality clean data, which were retained after these steps. At the same time, QC statistics including total reads number, raw data, raw depth, sequencing error rate and percentage of reads with Q30 (the percent of bases with phred-scaled quality scores greater than 30) were calculated and summarized.

**(5) Reads Mapping to Reference Sequence**

Valid sequencing data was mapped to the reference human genome (UCSC hg19) by Burrows-Wheeler Aligner (BWA) software ^9^ to get the original mapping results stored in BAM format. If one or one paired read(s) were mapped to multiple positions, the strategy adopted by BWA was to choose the most likely placement. If two or more most likely placements presented, BWA picked one randomly. Then, SAMtools ^10^ and Picard (<http://broadinstitute.github.io/picard/)> were used to sort BAM files and do duplicate marking, local realignment, and base quality recalibration to generate final BAM file for computation of the sequence coverage and depth. Mapping step was very difficult due to mismatches, including true mutation and sequencing error, and duplicates resulted from PCR amplification. These duplicate reads were uninformative and shouldn’t be considered as evidence for variants. We used Picard to mark these duplicates for follow up analysis.

**(6) Variant Calling**

Samtools ^10^ mpileup and bcftools were used to do variant calling and identify SNP, InDels. Control-FREEC ^11^ was utilized to do CNV detection, while Crest ^12^ was specialized for SV discovery.

**(7) Functional Annotation**

Functional annotation was very important because the link between genetic variations and diseases would be clarified in this step. ANNOVAR ^13^ was performed to do annotation for VCF (Variant Call Format) obtained in the previous effort. dbSNP, 1000 Genome and other related existing databases were applied to characterize the detected variants. Given to the significance of exonic variants, gene transcript annotation databases, such as Consensus CDS, RefSeq, Ensembl and UCSC, were also included to determine amino acid alternation. Annotation content contained the variant position, variant type, conservative prediction, etc. These annotation results would help to locate disease causal mutant. The details of annotation were exhibited in supplemented material.

**(8) Somatic Mutation Calling**

The somatic SNV was detected by muTect ^14^, the somatic InDel by Strelka ^15^, and the somatic structural variants (SV) by CREST ^12^. Control-FREEC was used to detect somatic CNV ^11^.

**Whole genome bisulfite sequencing**

**(1) DNA quantification and qualification**

Genomic DNA degradation and contamination was monitored on agarose gels. DNA purity was checked using the NanoPhotometer® spectrophotometer (IMPLEN, CA, USA). DNA concentration was measured using Qubit® DNA Assay Kit in Qubit® 2.0 Flurometer (Life Technologies, CA, USA).

**(2) Library preparation and quantification**

A total amount of 100 nanogram genomic DNA spiked with 0.5 ng lambda DNA were fragmented by sonication to 200-300bp with Covaris S220. These DNA fragments were treated with bisulfite using EZ DNA Methylation-GoldTM Kit (Zymo Research), and the library kit was Accel -NGS Methyl -Seq DNA Library Kit for illumina. Library concentration was quantified by Qubit® 2.0 Flurometer (Life Technologies, CA, USA) and quantitative PCR, and the insert size was assayed on Agilent Bioanalyzer 2100 system.

**(3) Quality control**

The library preparations were sequenced on an Illumina Hiseq XTen or Novaseq platform and 125 bp/150 bp paired -end reads were generated. Image analysis and base calling were performed with Illumina CASAVA pipeline, and finally 125bp/150bp paired-end reads were generated. First, we use FastQC (fastqc_v0.11.5) to perform basic statistics on the quality of the raw reads. Then, those reads sequences produced by the Illumina pipleline in FASTQ format were pre-processed through Trimmomatic (Trimmomatic-0.36) software use the parameter (SLIDINGWINDOW: 4:15 ; LEADING:3, TRAILING:3 ; ILLUMINACLIP: adapter.fa: 2: 30: 10 ; MINLEN:36) The remaining reads that passed all the filtering steps was counted as clean reads and all subsequent analyses were based on this. At last, we use FastQC to perform basic statistics on the quality of the clean data reads.

**(4) Reference data preparation before analysis**

Before the analysis, we prepare the reference data for the species we study, which includes the reference sequence fasta file, the annotation file in gtf format, the GO annotation file, the description file and the gene region file in bed format. As for the bed files, we predict repeats through RepeatMasker, followed by getting CGI track from a genome use cpg IslandExt.

**(5) Reads mapping to the reference genome**

Bismark software (version 0.16.3) ^16^ was used to perform alignments of bisulfite-treated reads to a reference genome (-X 700 --dovetail). The reference genome was firstly transformed into bisulfite-converted version (C-to-T and G-to-A converted) and then indexed using bowtie2 ^17^. Sequence reads were also transformed into fully bisulfite-converted versions (C-to-T and G-to-A converted) before they are aligned to similarly converted versions of the genome in a directional manner. Sequence reads that produce a unique best alignment from the two alignment processes (original top and bottom strand) are then compared to the normal genomic sequence and the methylation state of all cytosine positions in the read is inferred. The same reads that aligned to the same regions of genome were regarded as duplicated ones. The sequencing depth and coverage were summarized using deduplicated reads. The results of methylation extractor (bismark_methylation_extractor, - no_overlap) were transformed into bigWig format for visualization using IGV browser. The sodium bisulfite non-coversion rate was calculated as the percentage of cytosine sequenced at cytosine reference positions in the lambda genome.

**(6) Estimating methylation level**

To identify the methylation site, we modeled the sum Mc of methylated counts as a binomial (Bin) random variable with methylation rate r:

**mC ~ Bin (mC + umC * r)**

In order to calculate the methylation level of the sequence, we divided the sequence into multiple bins, with bin size is 10kb. The sum of methylated and unmethylated read counts in each window were calculated. Methylation level (ML) for each window or C site shows the fraction of methylated Cs, and is defined as:

**ML(C) = reads(mC) / [reads(mC) + reads(C)]**

Calculated ML was further corrected with the bisulfite non-conversion rate according to previous studies ^18^. Given the bisulfite nonconversion rate r, the corrected ML was estimated as:

**ML_(corrected)_ = (ML - r) / (l - r)**

**(7) Differentially methylated analysis**

Differentially methylated regions (DMRs) were identified using the DSS software ^19-21^. The core of DSS is a new dispersion shrinkage method for estimating the dispersion parameter from Gamma-Poisson or Beta-Binomial distributions. DSS possess three characteristics to detect DMRs. First, spatial correlation. Proper utilization of the information from neighboring Cytosine sites can help improve estimation of methylation levels at each Cytosine site, and hence improve DMR detection. Second, the read depth of the Cytosine sites provides information on precision that can be exploited to improve statistical tests for DMR detection. Finally, the variance among biological replicates provides information necessary for a valid statistical test to detect DMRs，when there is no biological replicate, DSS combining data from nearby Cytosine sites and using them as ‘pseudo-replicates’ to estimate biological variance at specific locations. According to the distribution of DMRs through the genome, we defined the genes related to DMRs as genes whose gene body region (from TSS to TES) or promoter region (upstream 2kb from the TSS) have an overlap with the DMRs.

**Real-time PCR**

Total RNA was isolated from cells using TRIzol reagent (Invitrogen) according to the standard protocol. Two micrograms of high-quality RNAs were processed directly to cDNA by reverse transcription with Superscript III (Invitrogen) following the manufacturer's instruction in a total volume of 50 μl. Primers for the genes tested in the present experiments were listed as below. Amplification reactions were done in 20 μl of the LightCycler-DNA Master SYBR Green I mix (Roche Applied Science) with 10 pmol primer, 2 mM MgCl2, 200 μM deoxynucleotide triphosphate mixture, 0.5 units Taq DNA polymerase, and universal buffer. All of the reactions were done in triplicate in an iCycler iQ system (Bio-Rad), and the thermal cycling conditions were as follows: 95°C for 3 min; 40 cycles of 95°C for 30 s, 58°C for 20 s, and 72°C for 30 s; 72°C for 10 min.

SCG2: Forward 5’-tgagacacagcagtggccag-3’; Rev. 5’-ccgaccacatcttcataggc-3’

SLC2A3: Fwd. 5’-gcaagtcaccgtgctagagc-3’; Rev. 5’-aaaccagggaatggggcctg-3’

CHI3L1: Fwd. 5’-cgatcacatcgacacctggg-3’; Rev. 5’-gctgtcaatggtgaccttcc-3’

PTX3: Fwd. 5’-tggactccatcccactgagg-3’; Rev. 5’-ttggtctcactggatgcacg-3’

PLAT: Fwd. 5’-gcaacatcagtcatggctgc-3’; Rev. 5’-gctgctgttccagttggtgc-3’

ICAM1: Fwd. 5’-ggagccaatttctcgtgccg-3’; Rev. 5’-tcggtcccttctgagacctc-3’

TMEM45A: Fwd. 5’-gaatcaactcctgggctggc-3’; Rev. 5’-gacttgaccgcaatagctcc-3’

STT3A: Fwd. 5’-tatctcccgatctgtggctg-3’; Rev. 5’-gcctaccagagagatgacgc-3’

β-actin: Fwd. 5’-GATCATTGCTCCTCCTGAGC-3’; Rev. 5’-ACTCCTGCTTGCTGATCCAC-3’.

**In vitro protein binding assay**

His-tagged recombinant human EPHA2 extracellular domain and recombinant human PDGF-AA homodimer were incubated together (molar concentration ratio for EPHA2 : PDGFA = 1:1) in PBS at room temperature for 3 hr with rotation. After incubation, the sample were subjected to immunoprecipitation using anti-His tag antibody and western blotting.

**Dual Drug Combination Assay**

Cells were seeded in 96-well plates at an initial density of 2 × 10^3^ per well. At each time point, cells were stained with 100 μl of sterile MTT (0.5 mg/ml) for 2 hours at 37°C, followed by removal of the culture medium and addition of 100 μl of dimethyl sulfoxide. Absorbance was measured at 570 nm, using 655 nm as the reference wavelength. All experiments were carried out in triplicate. Synergistic effects were determined by using the Chou-Talalay method to calculate the CI ^22^.

**Statistics**

All experiments were repeated at least three times and each treatment was set up in triplicate, unless specially indicated otherwise. Data are presented as mean±SD. The software used in this work included GraphPad Prism 5, String Version 11.0, and Cytoscape 3.7.1. TCGA_GBM mRNA expression database and RPPA database were downloaded from <https://xenabrowser.net>. High and low expression of genes were defined using median value. DAVID Bioinformatics Resources ^23^ and Gene Set Enrichment Assay (GSEA) (*P* < 0.05, FDR < 0.25) ^24^ were used to analyze data. The statistical significance was determined by Student's t test and P < 0.05 was considered statistically significant. Kaplan-Meier survival plot sand log-rank statistics were used to evaluate the survival of patients. Pearson rank correlation was used to analyze the relationship between different genes or proteins.

**References:**

1 Tan, J. *et al.* Capillary morphogenesis protein 2 is a novel prognostic biomarker and plays oncogenic roles in glioma. *J Pathol*. **245**, 160-171, (2018).

2 Lv, D. *et al.* Optimized dissociation protocol for isolating human glioma stem cells from tumorspheres via fluorescence-activated cell sorting. *Cancer Lett*. **377**, 105-115, (2016).

3 Kutner, R. H., Zhang, X. Y. & Reiser, J. Production, concentration and titration of pseudotyped HIV-1-based lentiviral vectors. *Nat Protoc*. **4**, 495-505, (2009).

4 Wang, Y. *et al.* The crosstalk of mTOR/S6K1 and Hedgehog pathways. *Cancer Cell*. **21**, 374-387, (2012).

5 Lee, D. F. *et al.* IKK beta suppression of TSC1 links inflammation and tumor angiogenesis via the mTOR pathway. *Cell*. **130**, 440-455, (2007).

6 Schwanhausser, B. *et al.* Global quantification of mammalian gene expression control. *Nature*. **473**, 337-342, (2011).

7 Wang, L. *et al.* DEGseq: an R package for identifying differentially expressed genes from RNA-seq data. *Bioinformatics*. **26**, 136-138, (2010).

8 Robinson, M. D., McCarthy, D. J. & Smyth, G. K. edgeR: a Bioconductor package for differential expression analysis of digital gene expression data. *Bioinformatics*. **26**, 139-140, (2010).

9 Li, H. & Durbin, R. Fast and accurate short read alignment with Burrows-Wheeler transform. *Bioinformatics*. **25**, 1754-1760, (2009).

10 Li, H. *et al.* The Sequence Alignment/Map format and SAMtools. *Bioinformatics*. **25**, 2078-2079, (2009).

11 Boeva, V. *et al.* Control-FREEC: a tool for assessing copy number and allelic content using next-generation sequencing data. *Bioinformatics*. **28**, 423-425, (2012).

12 Wang, J. *et al.* CREST maps somatic structural variation in cancer genomes with base-pair resolution. *Nat Methods*. **8**, 652-654, (2011).

13 Wang, K., Li, M. & Hakonarson, H. ANNOVAR: functional annotation of genetic variants from high-throughput sequencing data. *Nucleic Acids Res*. **38**, e164, (2010).

14 Cibulskis, K. *et al.* Sensitive detection of somatic point mutations in impure and heterogeneous cancer samples. *Nature biotechnology*. **31**, 213-219, (2013).

15 Saunders, C. T. *et al.* Strelka: accurate somatic small-variant calling from sequenced tumor-normal sample pairs. *Bioinformatics*. **28**, 1811-1817, (2012).

16 Krueger, F. & Andrews, S. R. Bismark: a flexible aligner and methylation caller for Bisulfite-Seq applications. *Bioinformatics*. **27**, 1571-1572, (2011).

17 Langmead, B. & Salzberg, S. L. Fast gapped-read alignment with Bowtie 2. *Nat Methods*. **9**, 357-359, (2012).

18 Lister, R. *et al.* Global epigenomic reconfiguration during mammalian brain development. *Science*. **341**, 1237905, (2013).

19 Feng, H., Conneely, K. N. & Wu, H. A Bayesian hierarchical model to detect differentially methylated loci from single nucleotide resolution sequencing data. *Nucleic Acids Res*. **42**, e69, (2014).

20 Wu, H. *et al.* Detection of differentially methylated regions from whole-genome bisulfite sequencing data without replicates. *Nucleic Acids Res*. **43**, e141, (2015).

21 Park, Y. & Wu, H. Differential methylation analysis for BS-seq data under general experimental design. *Bioinformatics*. **32**, 1446-1453, (2016).

22 Chou, T. C. Drug combination studies and their synergy quantification using the Chou-Talalay method. *Cancer Res*. **70**, 440-446, (2010).

23 Huang da, W., Sherman, B. T. & Lempicki, R. A. Systematic and integrative analysis of large gene lists using DAVID bioinformatics resources. *Nat Protoc*. **4**, 44-57, (2009).

24 Subramanian, A. *et al.* Gene set enrichment analysis: a knowledge-based approach for interpreting genome-wide expression profiles. *Proc Natl Acad Sci U S A*. **102**, 15545-15550, (2005).


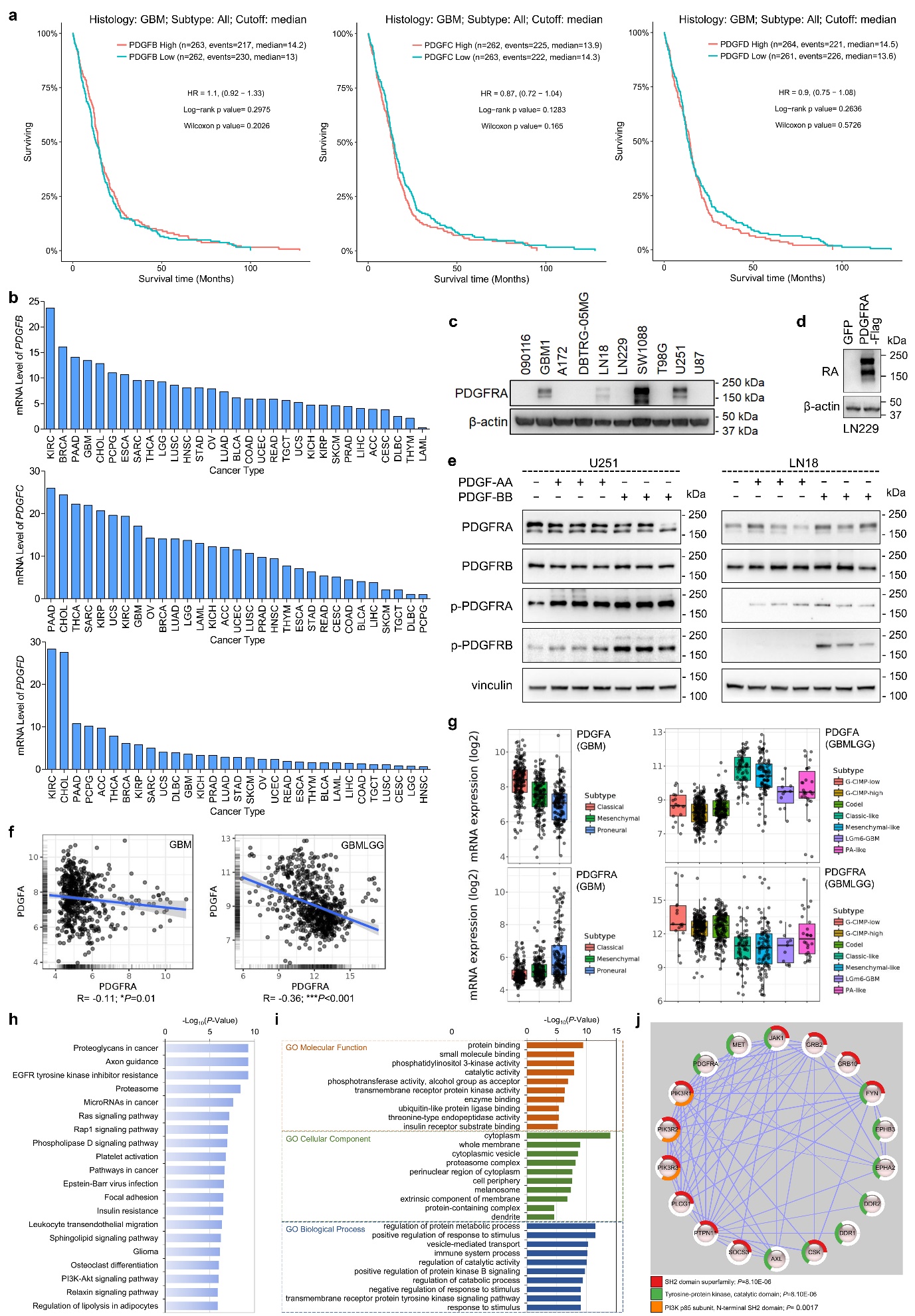


Figure. S1. Expression and function characteristics of PDGFA and PDGFRA in GBM.

**a)** Kaplan-Meier survival analysis of cases with PDGFB^High^ *vs.* PDGFB^Low^, PDGFC^High^ *vs.* PDGFC^Low^, or PDGFD^High^ *vs.* PDGFD^Low^ from TCGA_GBM database. **b)** PDGFB, PDGFC, or PDGFD gene expression in TCGA PanCancer databases. **c)** PDGFRA protein expression in a panel of glioma cell lines examined by western blotting. β-actin is used as loading control. **d)** PDGFRA expression by western blotting in LN229 cells stably transfected with GFP or PDGFRA-Flag. **e)** Regulation of PDGFRA and PDGFRB by PDGFA and PDGFB measured by western blotting. Vinculin is used as loading control. **f)** Pearson correlation of PDGFA and PDGFRA in TCGA_GBM and TCGA_GBMLGG databases. **g)** MRNA expression of PDGFA and PDGFRA in different molecular subtypes in TCGA_GBM and TCGA_GBMLGG databases. **h)** KEGG analysis of PDGFA-associated proteins. Top 20 genesets are listed in graph. **i)** GO analysis of PDGFA-associated proteins. Top 10 genesets for each subcategory are listed in graph. **j)** PDGFA-associated proteins involving SH2 domain and tyrosine kinase catalytic domain.


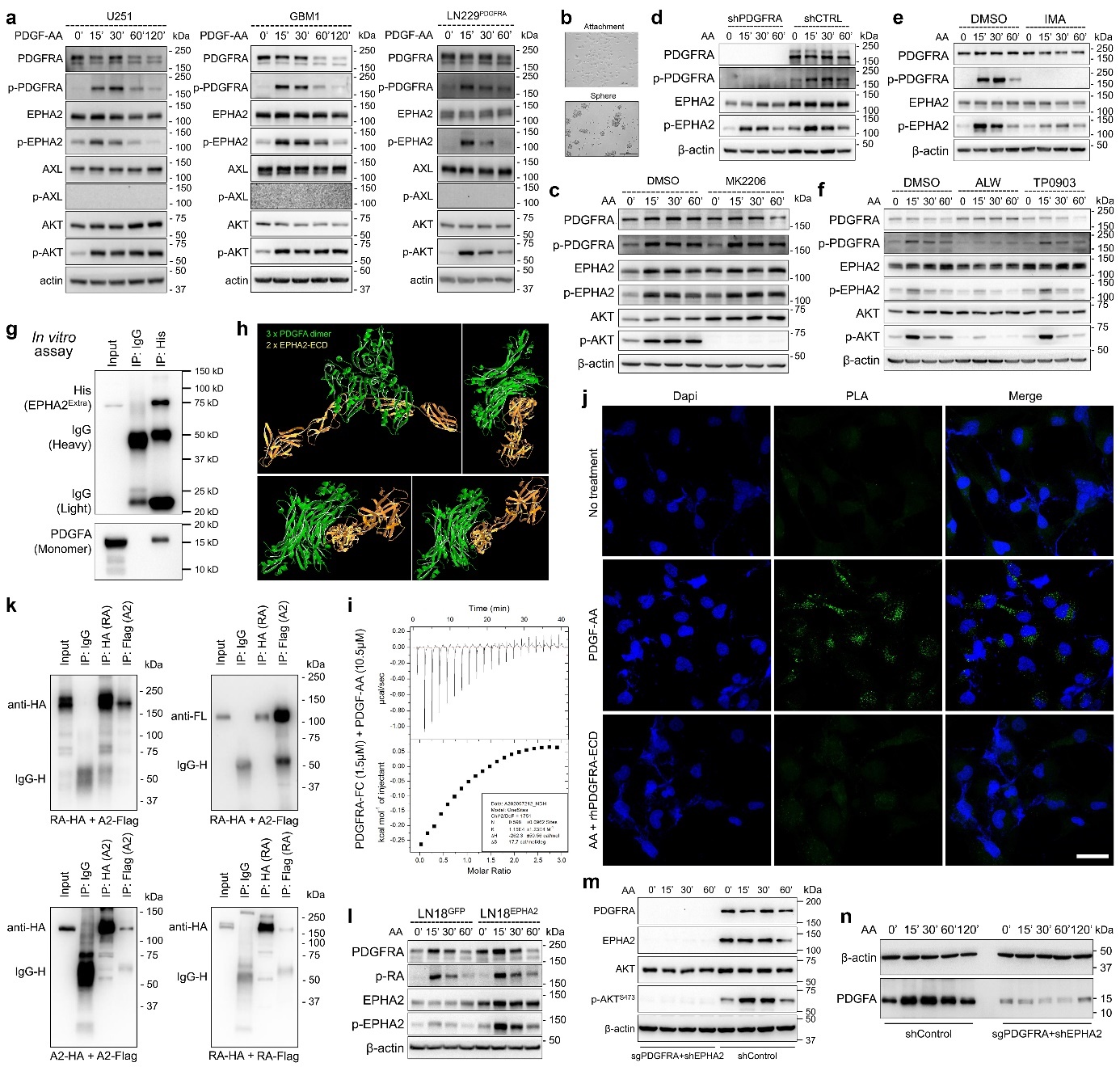
Figure. S2. Regulation of EPHA2 by PDGFA in GBM cells.

**a)** PDGFA-induced temporal expression of indicated proteins in GBM cells examined by western blotting. β-actin is used as loading control. **b)** Representative images of attachment and sphere culture of LN18 cells. **c)** PDGF-A-induced temporal expression of indicated proteins in U251 cells pre-treated with DMSO and MK2206. **b)** PDGFA-induced temporal expression of indicated proteins in U251 cells infected with lentivirus containing control shRNA or shRNA targeting PDGFRA. **e)** PDGFA-induced temporal expression of indicated proteins in U251 cells pre-treated with vehicle or IMA. **f)** PDGFA-induced temporal expression of indicated proteins in LN18 cells pre-treated with DMSO, EPHA2 inhibitor (ALW), and AXL inhibitor (TP0903). **g)** **h)** Interaction simulation of three dimension structure of PDGFA and EPHA2 extracellular domain. **i)** Interaction thermodynamics of recombinant human PDGFRA extracellular domain and recombinant human PDGF-AA using Microcal iTC200. **j)** Proximity ligation assay using U251 cells without treatment, treated with PDGFA for 15 min, or pre-treated with recombinant PDGFRA extracellular domain followed by PDGFA treatment for 15 min. The cells were counterstained with Dapi (blue) to mark nuclei. Green dot signals represent interaction between EPHA2 and PDGFA. Scale Bar = 25 μm. **k)** Co-immunoprecipitation and western blotting of tagged EPHA2 and tagged PDGFRA-HA in LN18 cells, which are transfected with two differently tagged EPHA2 plasmids, two differently tagged PDGFRA plasmids, or two differently tagged EPHA2 and PDGFRA plasmids, respectively. **l)** PDGFA-induced temporal expression of indicated proteins in LN18 cells transfected with GFP control or EPHA2. **m)** and **n)** PDGFA-induced temporal expression of indicated proteins in LN18 cells with deficiency of PDGFRA and EPHA2 *vs.* control shRNA.


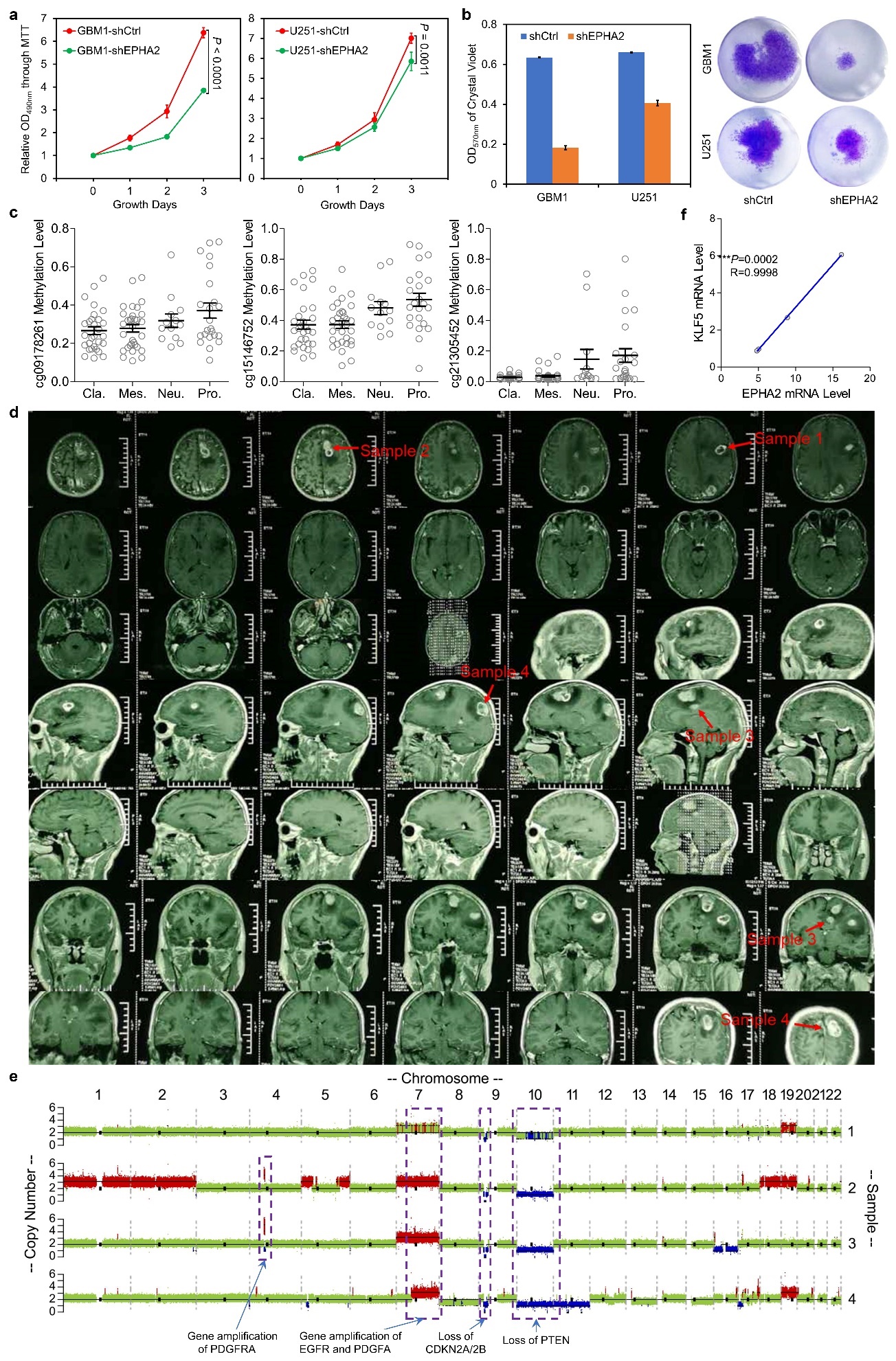


**Figure. S3. EPHA2 regulation in TCGA_GBM database and multifocal GBM.**

**a)** MTT assay of indicated cells indicated cells transfected with control shRNA or shRNA targeting EPHA2. **b)** Matrigel-coated transwell assay of indicated cells transfected with control shRNA or shRNA targeting EPHA2. **c)** Methylation levels of three methylation probes in different molecular subtypes of GBM in TCGA_GBM database. **d)** Series of MRI images of the patient with mGBM showing multi tumors labeled with red arrows and red font. **e)** Distribution features of chromosomal copy number variation of the four samples in four tumor foci from a multifocal GBM patient. Red color and blue color represent chromosomal gain and loss, respectively. **f)** Pearson correlation of EPHA2 and KLF5 in four tumor foci.


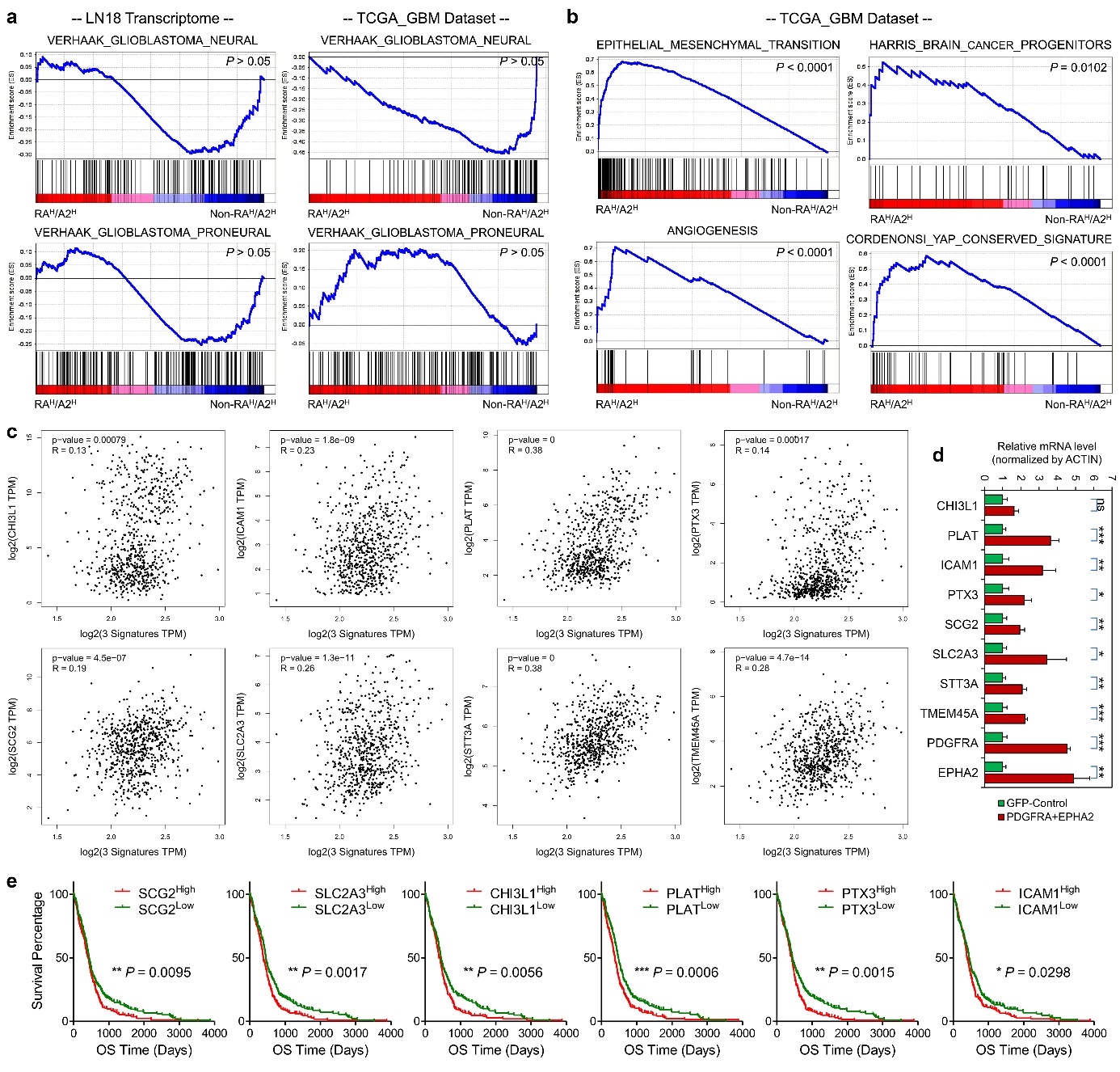


**Figure. S4. Transcriptomic analyses on PDGFRA and EPHA2 co-upregulation in GBM.**

**a)** Enrichment of signature genes of GBM molecular subtype for LN18 cells with co-transfection of EPHA2 and PDGFRA (RA^H^/A2^H^) *vs.* individual transfection (Non-RA^H^/A2^H^) (left two panels), as well as, cases with PDGFRA^High^/EPHA2^High^ (RA^H^/A2^H^) *vs.* all other (Non-RA^H^/A2^H^) cases from TCGA_GBM mRNA expression dataset (right two panels). **b)** Enrichment of signature genes of cancer-related features for cases with RA^H^/A2^H^ *vs.* Non-RA^H^/A2^H^ from TCGA_GBM mRNA expression dataset. **c)** Pearson correlation of significantly upregulated eight genes with combined expression of PDGFA, PDGFRA and EPHA2 (3 signature) using TCGA_GBM mRNA expression dataset. **d)** Real-time PCR of indicated genes using LN18 cells co-transfected with PDGFRA and EPHA2 *vs.* GFP control. **e)** Kaplan-Meier survival analysis of top altered genes consistently detected in LN18 cells with RA^H^/A2^H^ *vs.* Non-RA^H^/A2^H^ and cases with RA^H^/A2^H^ *vs.* Non-RA^H^/A2^H^ cases from TCGA_GBM mRNA expression dataset.


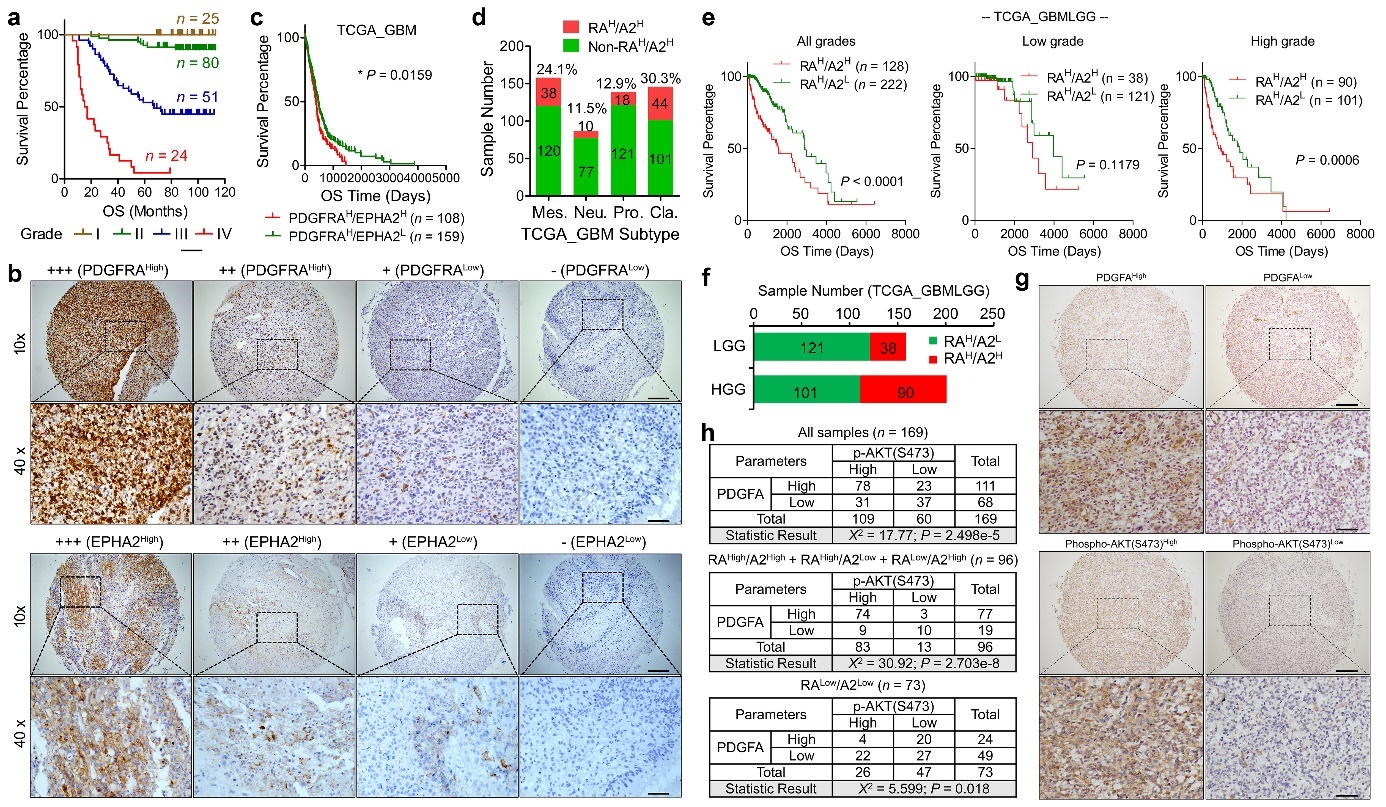


**Figure. S5. Clinical significance of EPHA2 and PDGFRA in GBM.**

**a)** Kaplan-Meier survival analysis on data from our glioma cohort according to tumor grades. **b)** Representative immunohistochemistry images of PDGFRA and EPHA2 protein with different expression intensity using glioma tissue microarray. Scale Bar = 200 μm (upper) and 50 μm (lower). **c)** Kaplan-Meier survival analysis on cases with *PDGFRA*^High^/EPHA2^High^ *vs.* PDGFRA^High^/EPHA2^Low^ from TCGA_GBM mRNA expression dataset. **d)** Case count with different protein expression patterns from TCGA_GBM mRNA expression dataset according to molecular subtypes. **e)** Kaplan-Meier survival analysis on cases with *PDGFRA*^High^/EPHA2^High^ *vs.* PDGFRA^High^/EPHA2^Low^ in all grades, high grade, and low grade, respectively, from TCGA_GBMLGG mRNA expression dataset. **f)** Case count with different protein expression patterns in high grade glioma (HGG) and low grade glioma (LGG) from TCGA_GBMLGG mRNA expression dataset. **g)** Representative immunohistochemistry images of PDGFA and phosphorylated AKT(S473) protein using glioma tissue microarray. Scale Bar = 200 μm (upper) and 50 μm (lower). **h)** *χ*^2^ test of different protein expression patterns using glioma tissue microarray.


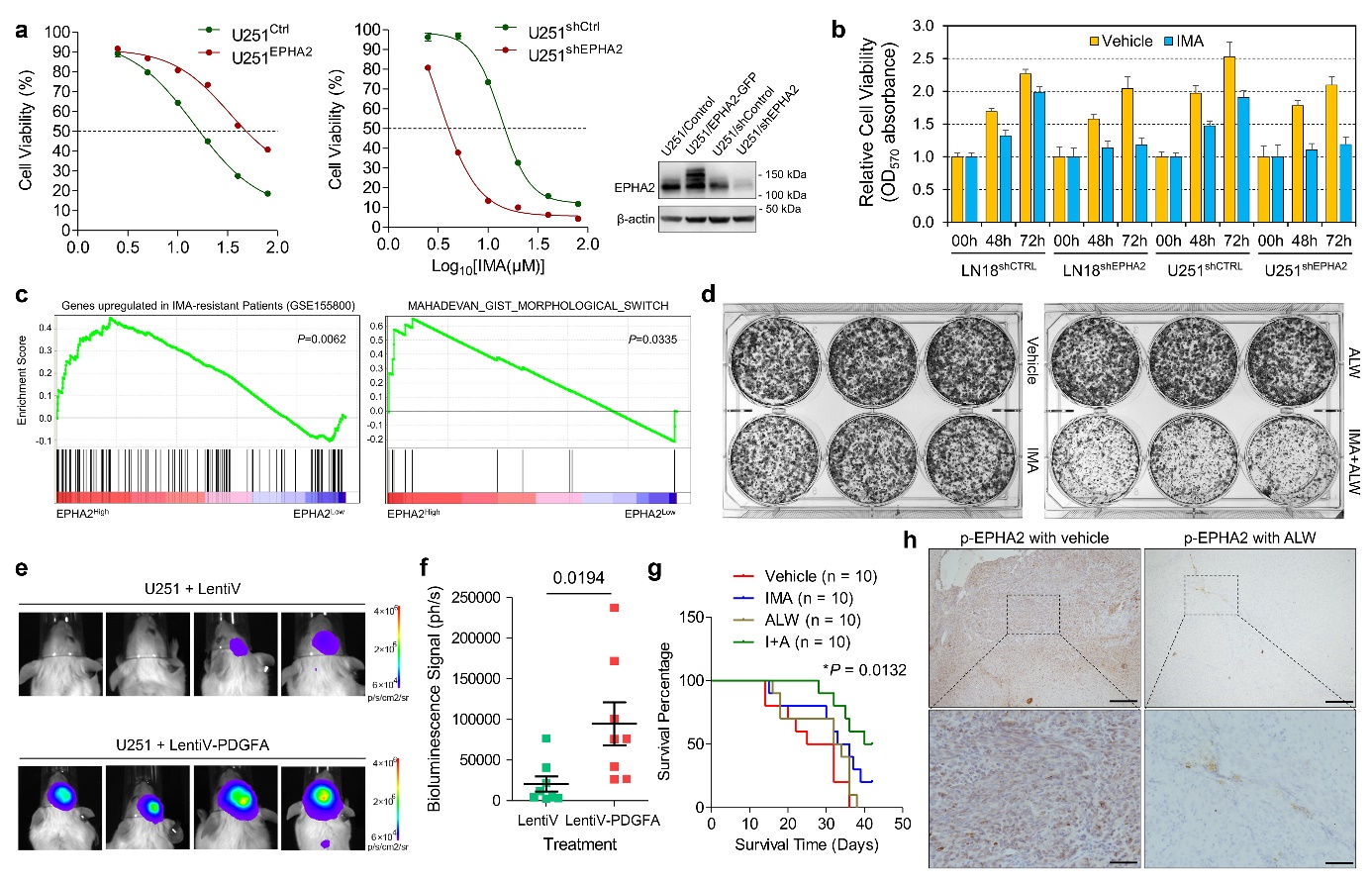


**Figure. S6. Increased sensitivity of GBM cells to IMA by knockdown of EPHA2 expression.**

**a)** IC_50_ measurement of U251 cells with forced expression of EPHA2 or knockdown of EPHA2 through MTT assay and western blotting confirmation. β-actin is used as loading control. **b)** Relative response of engineered GBM cells to vehicle and IMA. **c)** Enrichment of IMA resistance-related genes of GBM with EPHA2^High^ *vs.* EPHA2^Low^. **d)** Representative colony formation images of LN18 cells treated with vehicle, IMA (10μM), ALW (1μM), and IMA+ALW. **e)** Representative images of orthotopic growth of U251 cells treated with control lentivirus (LentiV) or lentivirus expressing PDGFA (LentiV-PDGFA). **f)** Survival curve of mice orthotopically inoculated with U251 cells and treated with Vehicle, IMA, ALW, or IMA + ALW. **h)** Representative immunohistochemistry images of phosphorylated EPHA2 protein in tumor sections from mice orthotopically inoculated with U251 cells and treated with Vehicle or ALW. Scale Bar = 200 μm (upper) and 50 μm (lower).

**Table S1. Protein categories of PDGFA interactome.**

| **Gene Symbol** | **Category** | **Gene Symbol** | **Category** | **Gene Symbol** | **Category** | **Gene Symbol** | **Category** |
| --- | --- | --- | --- | --- | --- | --- | --- |
| ERLIN2 | Lipid Metabolism | AXL | Kinase | NDFIP1 | Endosome | PPM1B | Hydrolase |
| CPT1A | Lipid Metabolism | EPHA2 | Kinase | NDFIP2 | Endosome | PTPN11 | Hydrolase |
| SPTLC1 | Lipid Metabolism | EPHB3 | Kinase | RAB9A | Endosome | VCP | Hydrolase |
| GM2A | Lipid Metabolism | FYN | Kinase | WDFY1 | Endosome | CNDP2 | Hydrolase |
| ACSL3 | Lipid Metabolism | JAK1 | Kinase | ARRDC3 | Endosome | CTSB | Hydrolase |
| PLCG1 | Lipid Metabolism | MET | Kinase | FLOT1 | Endosome | PSMA1 | Hydrolase |
| SLC27A4 | Lipid Metabolism | ACVR1B | Kinase | GRB2 | Endosome | PSMA2 | Hydrolase |
| TECR | Lipid Metabolism | ADK | Kinase | HGS | Endosome | PSMA7 | Hydrolase |
| DAAM2 | Cytoskeleton | BMPR2 | Kinase | ITM2B | Endosome | PSMB4 | Hydrolase |
| XIRP2 | Cytoskeleton | CSK | Kinase | ITGB1 | Endosome | PSMB5 | Hydrolase |
| PDLIM5 | Cytoskeleton | CSNK1A1 | Kinase | PMEPA1 | Endosome | PSMB6 | Hydrolase |
| MAP7D3 | Cytoskeleton | CSNK1D | Kinase | SNX17 | Endosome | PGAM5 | Hydrolase |
| RANGAP1 | Cytoskeleton | ddr1 | Kinase | SNX27 | Endosome | GANAB | Hydrolase |
| CCDC124 | Cytoskeleton | DDR2 | Kinase | STX12 | Endosome | PON2 | Hydrolase |
| CORO1B | Cytoskeleton | ERBB3 | Kinase | TMEM59 | Endosome | PPP1CA | Hydrolase |
| KIF11 | Cytoskeleton | PIK3CA | Kinase | VAMP7 | Endosome |  |  |
| KIF2A | Cytoskeleton | pik3cb | Kinase | LRP10 | Endosome | GRB10 | Unclassified |
| KIF2C | Cytoskeleton | PIK3R1 | Kinase | LRP12 | Endosome | NOTCH2 | Unclassified |
| STOML2 | Cytoskeleton | PIK3R2 | Kinase | TFRC | Endosome | TNFRSF10B | Unclassified |
| SDCBP | Cytoskeleton | PIK3R3 | Kinase | ARHGDIA | Ubl conjugation | UGDH | Unclassified |
| PLXNB2 | Cytoskeleton | PDGFRA | Kinase | CSDE1 | Ubl conjugation | BROX | Unclassified |
| SOCS3 | Cytoskeleton | PRKACB | Kinase | HIST1H1B | Ubl conjugation | ITM2C | Unclassified |
| STUB1 | Ligase | TGFBR2 | Kinase | ITCH | Ubl conjugation | KIRREL | Unclassified |
| DARS2 | Ligase | ABCA13 | Transport | PSMC4 | Ubl conjugation | PABPC3 | Unclassified |
| KARS | Ligase | ATP6AP1 | Transport | PSMD1 | Ubl conjugation | C1orf27 | Unclassified |
| RNF114 | Ligase | LMAN1 | Transport | PSMD11 | Ubl conjugation | CD320 | Unclassified |
| RNF128 | Ligase | SLC12A2 | Transport | PSMD3 | Ubl conjugation | FAM129A | Unclassified |
| RNF130 | Ligase | SLC20A1 | Transport | RPN2 | Ubl conjugation | GALE | Unclassified |
| RNF149 | Ligase | SLC26A2 | Transport | SKP1 | Ubl conjugation | IGLL5 | Unclassified |
| SUCLG1 | Ligase | SLC30A1 | Transport | SYT11 | Ubl conjugation | LIMS1 | Unclassified |
| TRIM22 | Ligase | STX10 | Transport | UBB | Ubl conjugation | mocs2 | Unclassified |
| CTPS1 | Ligase | STX4 | Transport | USP8 | Ubl conjugation | PGM3 | Unclassified |
| NQO1 | NADP | STX6 | Transport | FAF2 | Ubl conjugation | rbm12 | Unclassified |
| AKR1B1 | NADP | STXBP3 | Transport | UBXN1 | Ubl conjugation | TIPRL | Unclassified |
| AKR7A2 | NADP | CST1 | Protease inhibitor | CD44 | Cell adhesion | TNFRSF1A | Unclassified |
| DHRS3 | NADP | SERPINE2 | Protease inhibitor | ADGRE5 | Cell adhesion | TPMT | Unclassified |
| RDH10 | NADP | TIMP3 | Protease inhibitor | DSG2 | Cell adhesion | SEPTIN8 | Unclassified |
| ZADH2 | NADP | BNIP3 | mitochondria | PCDH1 | Cell adhesion | c19orf53 | Unclassified |
| BAG2 | Chaperon | FKBP8 | mitochondria | PCDHGC3 | Cell adhesion | CRIP2 | Unclassified |
| DNAJA2 | Chaperon | ACAT1 | mitochondria | THBS1 | Cell adhesion | GNB2 | Unclassified |
| DNAJA3 | Chaperon | CS | mitochondria | GNAI2 | GTP-binding | GPX1 | Unclassified |
| DNAJB6 | Chaperon | C1QBP | mitochondria | gnai3 | GTP-binding | IFIT5 | Unclassified |
| DNAJC7 | Chaperon | ETFB | mitochondria | EIF5 | GTP-binding | KPRP | Unclassified |
| CANX | Chaperon | SHMT2 | mitochondria | RHOA | GTP-binding | KRT72 | Unclassified |
| CCT4 | Chaperon | Uqcrfs1 | mitochondria | RRAS2 | GTP-binding | NUCKS1 | Unclassified |
| HSP90AA1 | Chaperon | VDAC3 | mitochondria | GPRC5A | GTP-binding | NUDCD1 | Unclassified |
| TMEM33 | nuclear envelope | EMD | nuclear envelope | RRM1 | nuclear envelope | TMCC1 | Unclassified |

**Table S2. List of transcription factors significantly correlated with EPHA2 in TCGA_GBM database.**

| **Gene** | **name** | **r** | **p.value** | **adj.p.value** |
| --- | --- | --- | --- | --- |
| KLF5 | Kruppel like factor 5 | 0.417 | 0 | 0 |
| TEAD3 | TEA domain transcription factor 3 | 0.412 | 0 | 0 |
| ETV4 | ETS variant 4 | 0.408 | 0 | 0 |
| YAP1 | Yes associated protein 1 | 0.382 | 0 | 0 |
| HDAC1 | histone deacetylase 1 | 0.38 | 0 | 0 |
| ERBB2 | erb-b2 receptor tyrosine kinase 2 | 0.379 | 0 | 0 |
| PHF10 | PHD finger protein 10 | 0.363 | 0 | 0 |
| TGIF1 | TGFB induced factor homeobox 1 | 0.36 | 0 | 0 |
| HMGA2 | high mobility group AT-hook 2 | 0.354 | 0 | 0 |
| ELK3 | ELK3, ETS transcription factor | 0.353 | 0 | 0 |
| ELF3 | E74 like ETS transcription factor 3 | 0.346 | 0 | 0 |
| ELF4 | E74 like ETS transcription factor 4 | 0.345 | 2.22E-16 | 2.82E-12 |
| VDR | vitamin D (1,25- dihydroxyvitamin D3) receptor | 0.343 | 2.22E-16 | 2.82E-12 |
| PML | promyelocytic leukemia | 0.341 | 4.44E-16 | 5.64E-12 |
| SMAD3 | SMAD family member 3 | 0.341 | 4.44E-16 | 5.64E-12 |
| ZBED2 | zinc finger BED-type containing 2 | 0.327 | 7.55E-15 | 9.59E-11 |
| TEAD4 | TEA domain transcription factor 4 | 0.325 | 1.15E-14 | 1.47E-10 |
| GATA6 | GATA binding protein 6 | 0.322 | 2.07E-14 | 2.62E-10 |
| FHL2 | four and a half LIM domains 2 | 0.32 | 2.93E-14 | 3.72E-10 |
| GRHL2 | grainyhead like transcription factor 2 | 0.319 | 3.66E-14 | 4.65E-10 |
| NPAS2 | neuronal PAS domain protein 2 | 0.316 | 6.39E-14 | 8.12E-10 |
| MDFIC | MyoD family inhibitor domain containing | 0.315 | 7.73E-14 | 9.81E-10 |
| PITX1 | paired like homeodomain 1 | 0.31 | 1.77E-13 | 2.25E-09 |
| PAWR | pro-apoptotic WT1 regulator | 0.309 | 2.49E-13 | 3.16E-09 |
| IRF6 | interferon regulatory factor 6 | 0.307 | 3.03E-13 | 3.85E-09 |
| BCL3 | B-cell CLL/lymphoma 3 | 0.303 | 7.44E-13 | 9.44E-09 |
| KANK2 | KN motif and ankyrin repeat domains 2 | 0.301 | 1.03E-12 | 1.31E-08 |
| PBX1 | PBX homeobox 1 | -0.301 | 9.9E-13 | 1.26E-08 |
| INSM1 | INSM transcriptional repressor 1 | -0.302 | 7.74E-13 | 9.84E-09 |
| ZHX2 | zinc fingers and homeoboxes 2 | -0.305 | 4.74E-13 | 6.02E-09 |
| APBB1 | amyloid beta precursor protein binding family B member 1 | -0.305 | 4.4E-13 | 5.59E-09 |
| ASH2L | ASH2 like histone lysine methyltransferase complex subunit | -0.309 | 2.24E-13 | 2.85E-09 |
| HEY2 | hes related family bHLH transcription factor with YRPW motif 2 | -0.309 | 2.24E-13 | 2.84E-09 |
| ZEB1 | zinc finger E-box binding homeobox 1 | -0.31 | 2.01E-13 | 2.56E-09 |
| ARNT2 | aryl hydrocarbon receptor nuclear translocator 2 | -0.313 | 1.14E-13 | 1.45E-09 |
| ASCL1 | achaete-scute family bHLH transcription factor 1 | -0.315 | 7.02E-14 | 8.92E-10 |
| VAX2 | ventral anterior homeobox 2 | -0.322 | 1.91E-14 | 2.42E-10 |
| ZEB2 | zinc finger E-box binding homeobox 2 | -0.332 | 2.84E-15 | 3.6E-11 |
| MEF2C | myocyte enhancer factor 2C | -0.34 | 5.16E-16 | 6.55E-12 |
| DPF1 | double PHD fingers 1 | -0.342 | 3.27E-16 | 4.15E-12 |
| ID2 | inhibitor of DNA binding 2, HLH protein | -0.354 | 2.41E-17 | 3.06E-13 |
| MYT1 | myelin transcription factor 1 | -0.357 | 1.32E-17 | 1.68E-13 |
| SOX10 | SRY-box 10 | -0.366 | 1.55E-18 | 1.96E-14 |
| OLIG2 | oligodendrocyte lineage transcription factor 2 | -0.374 | 2.51E-19 | 3.19E-15 |
| MXI1 | MAX interactor 1, dimerization protein | -0.379 | 7.8E-20 | 9.9E-16 |
| SATB1 | SATB homeobox 1 | -0.395 | 1.65E-21 | 2.09E-17 |

**Table S3. Pearson correlation of methylation levels in EPHA2 promoter region with EPHA2 mRNA expression in TCGA_GBM database.**

| **Methylation K450 Probe ID** | **gene** | **GRCh37 Chrom** | **Chrom Start** | **Chrom End** | **Location towards transcription start site** | **Number of XY Pairs** | **r** | **P value (two-tailed)** | **Signifi-cance** |
| --- | --- | --- | --- | --- | --- | --- | --- | --- | --- |
| cg21305452 | RP11-276H7.2,EPHA2 | chr1 | 16481936 | 16481938 | 644 | 85 | -0.2416 | 0.0259 | * |
| cg16886895 | RP11-276H7.2,EPHA2 | chr1 | 16481989 | 16481991 | 591 | 85 | -0.1046 | 0.3408 | ns |
| cg08137080 | RP11-276H7.2,EPHA2 | chr1 | 16482429 | 16482431 | 151 | 85 | -0.0923 | 0.4008 | ns |
| cg15483964 | RP11-276H7.2,EPHA2 | chr1 | 16482438 | 16482440 | 142 | 84 | -0.08629 | 0.4351 | ns |
| cg15415507 | RP11-276H7.2,EPHA2 | chr1 | 16482552 | 16482554 | 28 | 85 | -0.06878 | 0.5316 | ns |
| cg01495321 | RP11-276H7.2,EPHA2 | chr1 | 16482605 | 16482607 | -25 | 85 | -0.1539 | 0.1597 | ns |
| cg15146752 | RP11-276H7.2,EPHA2 | chr1 | 16482766 | 16482768 | -186 | 85 | -0.2572 | 0.0175 | * |
| cg09178261 | RP11-276H7.2,EPHA2 | chr1 | 16482817 | 16482819 | -237 | 85 | -0.2381 | 0.0282 | * |
| cg26793227 | RP11-276H7.2,EPHA2 | chr1 | 16483657 | 16483659 | -1077 | 84 | -0.07435 | 0.5015 | ns |
| cg16254190 | RP11-276H7.2,EPHA2 | chr1 | 16483693 | 16483695 | -1113 | 85 | 0.06417 | 0.5596 | ns |

**Table S4. Pearson correlation of EPHA2 with transcription factors listed in Table S2.**

| **Parameter** | **Number of XY Pairs** | **Pearson r** | **P value (two-tailed)** | **P value summary** |
| --- | --- | --- | --- | --- |
| KLF5 | 5 | 0.9828 | 0.0027 | ** |
| TEAD4 | 5 | 0.9652 | 0.0078 | ** |
| ELK3 | 5 | 0.9538 | 0.0118 | * |
| YAP1 | 5 | 0.9394 | 0.0177 | * |
| PAWR | 5 | 0.8966 | 0.0393 | * |
| NPAS2 | 5 | 0.8933 | 0.0412 | * |
| FHL2 | 5 | 0.8807 | 0.0486 | * |
| HMGA2 | 5 | 0.8665 | 0.0573 | ns |
| PITX1 | 5 | 0.8664 | 0.0574 | ns |
| VDR | 5 | 0.8635 | 0.0593 | ns |
| PML | 5 | 0.8529 | 0.0662 | ns |
| ASH2L | 5 | 0.7941 | 0.1087 | ns |
| MDFIC | 5 | 0.7718 | 0.1262 | ns |
| BCL3 | 5 | 0.7272 | 0.1638 | ns |
| ELF3 | 5 | 0.682 | 0.2047 | ns |
| ERBB2 | 5 | 0.6801 | 0.2064 | ns |
| TEAD3 | 5 | 0.6641 | 0.2215 | ns |
| SMAD3 | 5 | 0.6509 | 0.2342 | ns |
| ETV4 | 5 | 0.5012 | 0.3897 | ns |
| ZHX2 | 5 | 0.493 | 0.3987 | ns |
| HDAC1 | 5 | 0.4584 | 0.4375 | ns |
| KANK2 | 5 | 0.4579 | 0.4381 | ns |
| ZEB1 | 5 | 0.4471 | 0.4504 | ns |
| MEF2C | 5 | 0.4291 | 0.4709 | ns |
| GATA6 | 5 | 0.4179 | 0.4839 | ns |
| APBB1 | 5 | 0.4148 | 0.4874 | ns |
| PHF10 | 5 | 0.3998 | 0.5049 | ns |
| TGIF1 | 5 | 0.3795 | 0.5287 | ns |
| ELF4 | 5 | -0.3694 | 0.5406 | ns |
| HEY2 | 5 | 0.3626 | 0.5487 | ns |
| GRHL2 | 5 | -0.3581 | 0.554 | ns |
| PBX1 | 5 | 0.3237 | 0.5952 | ns |
| ARNT2 | 5 | 0.2645 | 0.6672 | ns |
| ID2 | 5 | 0.2624 | 0.6698 | ns |
| IRF6 | 5 | 0.2595 | 0.6733 | ns |
| DPF1 | 5 | 0.2446 | 0.6917 | ns |
| SOX10 | 5 | 0.2288 | 0.7112 | ns |
| SATB1 | 5 | 0.2137 | 0.73 | ns |
| MXI1 | 5 | 0.2029 | 0.7434 | ns |
| MYT1 | 5 | -0.1799 | 0.7722 | ns |
| ZEB2 | 5 | 0.1426 | 0.8191 | ns |
| OLIG2 | 5 | 0.1319 | 0.8325 | ns |
| INSM1 | 5 | 0.1217 | 0.8455 | ns |
| ASCL1 | 5 | 0.09421 | 0.8802 | ns |
| VAX2 | 5 | 0.07156 | 0.909 | ns |

**Table S5. Consistently altered genes in transfected LN18 transcriptome and TCGA_GBM database.**

| **Gene Symbol** | **Transfected LN18 cells** | | | **TCGA_GBM database** | | |
| --- | --- | --- | --- | --- | --- | --- |
|  | **RA^H^/A2^H^** | **non-RA^H^/A2^H^** | **Ratio** | **RA^H^/A2^H^** | **non-RA^H^/A2^H^** | **Ratio** |
| SCG2 | 4.56 | 2.79 | 1.63 | 9.49 | 8.95 | 1.06 |
| TMEM45A | 5.35 | 3.29 | 1.63 | 8.42 | 7.9 | 1.07 |
| SLC2A3 | 9.23 | 5.71 | 1.62 | 7.2 | 6.8 | 1.06 |
| CHI3L1 | 14.57 | 9.09 | 1.6 | 11.82 | 11.05 | 1.07 |
| PLAT | 32.16 | 20.16 | 1.59 | 9.06 | 8.42 | 1.08 |
| PTX3 | 56.51 | 36.68 | 1.54 | 8.59 | 7.64 | 1.12 |
| ICAM1 | 7.24 | 4.72 | 1.54 | 5.83 | 5.53 | 1.05 |
| STT3A | 72.83 | 48.49 | 1.5 | 7.36 | 6.88 | 1.07 |
| ANPEP | 9.92 | 6.64 | 1.49 | 4.87 | 4.46 | 1.09 |
| POSTN | 59.23 | 40.14 | 1.48 | 9.34 | 7.91 | 1.18 |
| ENG | 52.9 | 35.93 | 1.47 | 6.01 | 5.68 | 1.06 |
| MLC1 | 6.48 | 4.49 | 1.44 | 8.52 | 8.1 | 1.05 |
| LAMA4 | 16.28 | 11.37 | 1.43 | 6.46 | 5.96 | 1.08 |
| MXRA8 | 12.81 | 8.96 | 1.43 | 7.46 | 6.9 | 1.08 |
| NRP1 | 6.32 | 4.42 | 1.43 | 5.82 | 5.53 | 1.05 |
| SOAT1 | 10.73 | 7.59 | 1.41 | 6.4 | 5.92 | 1.08 |
| EDNRA | 8.91 | 6.32 | 1.41 | 7.18 | 6.53 | 1.1 |
| PDGFRB | 21.48 | 15.27 | 1.41 | 7.12 | 6.66 | 1.07 |
| SRPX2 | 5.8 | 4.14 | 1.4 | 7.12 | 6.27 | 1.13 |
| PTGS1 | 81.69 | 58.45 | 1.4 | 6.4 | 6.01 | 1.06 |
| TCN2 | 5.74 | 4.11 | 1.4 | 6.4 | 6.04 | 1.06 |
| GAA | 18.73 | 13.45 | 1.39 | 7.01 | 6.66 | 1.05 |
| COL4A1 | 99.91 | 71.71 | 1.39 | 10.02 | 9.04 | 1.11 |
| VCAM1 | 18.7 | 13.44 | 1.39 | 8.71 | 8.18 | 1.06 |
| CA12 | 1.61 | 1.16 | 1.39 | 7.77 | 7.19 | 1.08 |
| ACVR1 | 10.68 | 7.69 | 1.39 | 7.94 | 7.55 | 1.05 |
| COL3A1 | 233.97 | 168.76 | 1.39 | 9.69 | 8.62 | 1.12 |
| BMP1 | 16.67 | 12.05 | 1.38 | 5.69 | 5.34 | 1.07 |
| PLAU | 285.42 | 206.6 | 1.38 | 6.31 | 5.85 | 1.08 |
| ITGA5 | 120.13 | 87.19 | 1.38 | 7.58 | 7.01 | 1.08 |
| RHBDF1 | 52.24 | 38.18 | 1.37 | 7.1 | 6.66 | 1.07 |
| TNC | 18.72 | 13.77 | 1.36 | 10.67 | 9.92 | 1.08 |
| P4HA2 | 29.5 | 21.72 | 1.36 | 7.02 | 6.59 | 1.07 |
| FN1 | 87.1 | 64.17 | 1.36 | 9.65 | 9.18 | 1.05 |
| COL6A2 | 227.65 | 168 | 1.36 | 6.53 | 5.72 | 1.14 |
| SLC2A10 | 21.9 | 16.2 | 1.35 | 7.89 | 7.29 | 1.08 |
| LTBP1 | 74.61 | 55.29 | 1.35 | 7.81 | 7.28 | 1.07 |
| SEZ6L2 | 6.41 | 4.76 | 1.35 | 6.9 | 6.47 | 1.07 |
| TNFSF10 | 10.26 | 7.63 | 1.34 | 6.98 | 6.62 | 1.05 |
| THBS3 | 5.55 | 4.14 | 1.34 | 6.21 | 5.89 | 1.05 |
| TFPI | 11.04 | 8.24 | 1.34 | 6.21 | 5.85 | 1.06 |
| PCOLCE | 38.96 | 29.1 | 1.34 | 7.75 | 6.8 | 1.14 |
| FKBP10 | 213.9 | 159.99 | 1.34 | 6.17 | 5.86 | 1.05 |
| VEGFA | 22.93 | 17.16 | 1.34 | 8.43 | 8 | 1.05 |
| MRC2 | 40.83 | 30.57 | 1.34 | 8.03 | 7.5 | 1.07 |
| OSMR | 46.28 | 34.67 | 1.33 | 5.88 | 5.55 | 1.06 |
| ECM1 | 10.6 | 7.95 | 1.33 | 6.42 | 6.07 | 1.06 |
| COL4A2 | 117.09 | 87.82 | 1.33 | 9.64 | 8.65 | 1.11 |
| NID1 | 55.78 | 41.84 | 1.33 | 7.72 | 6.76 | 1.14 |
| VLDLR | 2.66 | 2 | 1.33 | 6.2 | 5.82 | 1.06 |
| TGFB2 | 12.35 | 9.26 | 1.33 | 5.06 | 4.82 | 1.05 |
| COL1A2 | 16.61 | 12.46 | 1.33 | 10.37 | 9.36 | 1.11 |
| PLOD1 | 170.05 | 127.81 | 1.33 | 8.52 | 8 | 1.06 |
| ZNF606 | 2.86 | 2.15 | 1.33 | 7.26 | 6.79 | 1.07 |
| TM9SF4 | 47.92 | 36.17 | 1.32 | 7.03 | 6.63 | 1.06 |
| JAG1 | 45.37 | 34.27 | 1.32 | 7.74 | 7.23 | 1.07 |
| COL5A1 | 87.06 | 65.89 | 1.32 | 6.85 | 5.88 | 1.16 |
| NCSTN | 71.33 | 53.99 | 1.32 | 7.99 | 7.6 | 1.05 |
| EGFR | 20.38 | 15.45 | 1.32 | 7.67 | 7.15 | 1.07 |
| C8orf4 | 8.05 | 6.11 | 1.32 | 7.36 | 6.94 | 1.06 |
| MAN2B1 | 25.42 | 19.3 | 1.32 | 7.59 | 7.22 | 1.05 |
| SLC12A2 | 6.35 | 4.85 | 1.31 | 6 | 5.65 | 1.06 |
| PLXNA3 | 24.82 | 18.97 | 1.31 | 5.72 | 5.41 | 1.06 |
| COL5A2 | 108.06 | 82.6 | 1.31 | 9 | 7.97 | 1.13 |
| SLC17A5 | 6 | 4.6 | 1.31 | 6.86 | 6.53 | 1.05 |
| TSPAN4 | 19.62 | 15.02 | 1.31 | 6.31 | 5.94 | 1.06 |
| TIE1 | 2.44 | 1.87 | 1.31 | 5.44 | 5.03 | 1.08 |
| IQGAP2 | 2.33 | 1.79 | 1.3 | 6.96 | 6.53 | 1.07 |
| SLC5A6 | 21.98 | 16.91 | 1.3 | 7.11 | 6.77 | 1.05 |
| LAMA5 | 32.5 | 25 | 1.3 | 7.48 | 7.12 | 1.05 |
| HOXD10 | 13.17 | 10.14 | 1.3 | 4.62 | 4.37 | 1.06 |
| MMP2 | 90.04 | 69.42 | 1.3 | 8.33 | 7.56 | 1.1 |
| SLC27A3 | 14.16 | 10.92 | 1.3 | 7.04 | 6.6 | 1.07 |
| CRISPLD2 | 3.26 | 2.52 | 1.29 | 6.62 | 6.15 | 1.08 |
| FZD4 | 22.05 | 17.05 | 1.29 | 6.41 | 6.05 | 1.06 |
| NUCB1 | 62.23 | 48.13 | 1.29 | 7.7 | 7.26 | 1.06 |
| STC1 | 145.11 | 112.56 | 1.29 | 5.61 | 5.28 | 1.06 |
| TNFAIP3 | 9.32 | 7.23 | 1.29 | 7.06 | 6.67 | 1.06 |
| CDH6 | 9.34 | 7.24 | 1.29 | 5.17 | 4.9 | 1.06 |
| QSOX1 | 73.21 | 56.89 | 1.29 | 6.53 | 6.18 | 1.06 |
| LRP1 | 5.97 | 4.64 | 1.29 | 7.32 | 6.88 | 1.06 |
| EMILIN1 | 121.94 | 94.99 | 1.28 | 5.35 | 4.82 | 1.11 |
| COL9A2 | 25.72 | 20.04 | 1.28 | 6.12 | 5.72 | 1.07 |
| COL6A3 | 290.88 | 226.8 | 1.28 | 8.02 | 7.17 | 1.12 |
| MX2 | 1.84 | 1.43 | 1.28 | 6.17 | 5.81 | 1.06 |
| LAMB2 | 107.25 | 83.74 | 1.28 | 7.6 | 7.21 | 1.05 |
| FBLN1 | 12.76 | 9.96 | 1.28 | 5.37 | 5.06 | 1.06 |
| SERPINE1 | 77.68 | 60.7 | 1.28 | 7.69 | 7.17 | 1.07 |
| FZD6 | 9.59 | 7.5 | 1.28 | 5.92 | 5.46 | 1.08 |
| NOTCH1 | 2.61 | 2.04 | 1.28 | 7.7 | 7.33 | 1.05 |
| TMEM115 | 50.51 | 39.49 | 1.28 | 6.82 | 6.5 | 1.05 |
| ATP13A1 | 29.71 | 23.26 | 1.28 | 7.57 | 7.08 | 1.07 |
| BGN | 28.69 | 22.52 | 1.27 | 6.92 | 6.34 | 1.09 |
| LTBP2 | 2.54 | 1.99 | 1.27 | 6.9 | 6.57 | 1.05 |
| HRH1 | 6.32 | 4.96 | 1.27 | 7.31 | 6.93 | 1.05 |
| TGFBI | 148.65 | 116.93 | 1.27 | 10.38 | 9.76 | 1.06 |
| TIMP1 | 450.06 | 354.37 | 1.27 | 11.71 | 11.1 | 1.06 |
| PLAUR | 27.8 | 21.91 | 1.27 | 6.66 | 6.26 | 1.07 |
| EMP1 | 16.66 | 13.14 | 1.27 | 9.68 | 9.14 | 1.06 |
| SERPINH1 | 167.28 | 131.99 | 1.27 | 8.45 | 7.59 | 1.11 |
| ABCB1 | 10.93 | 8.65 | 1.26 | 5.67 | 5.3 | 1.07 |
| FGFR1 | 11.25 | 8.9 | 1.26 | 6.13 | 5.82 | 1.05 |
| SLC26A2 | 9.05 | 7.17 | 1.26 | 7.69 | 7.32 | 1.05 |
| FZD7 | 31.32 | 24.84 | 1.26 | 7.7 | 7.03 | 1.09 |
| KDELR1 | 95.46 | 75.82 | 1.26 | 7.55 | 7.04 | 1.07 |
| DPP4 | 15.62 | 12.41 | 1.26 | 4.95 | 4.65 | 1.07 |
| CDH11 | 1.28 | 1.02 | 1.25 | 9.21 | 8.63 | 1.07 |
| MEGF8 | 17.69 | 14.1 | 1.25 | 5.83 | 5.54 | 1.05 |
| FZD2 | 25.25 | 20.15 | 1.25 | 5.95 | 5.46 | 1.09 |
| ATP10D | 10.76 | 8.59 | 1.25 | 7.7 | 7.21 | 1.07 |
| CLPTM1 | 38.8 | 31.01 | 1.25 | 7.14 | 6.8 | 1.05 |
| SRPX | 15.37 | 12.29 | 1.25 | 10.23 | 9.54 | 1.07 |
| LAMB1 | 308.22 | 246.71 | 1.25 | 8.02 | 7.07 | 1.13 |
| PDIA4 | 203.75 | 163.21 | 1.25 | 8.08 | 7.62 | 1.06 |
| IGFBP2 | 9.92 | 7.95 | 1.25 | 9.78 | 8.82 | 1.11 |
| SLC4A3 | 1.43 | 1.15 | 1.25 | 6.24 | 5.89 | 1.06 |
| PDGFRL | 3.94 | 3.16 | 1.25 | 5.71 | 5.38 | 1.06 |
| NEO1 | 24.69 | 19.83 | 1.24 | 6.76 | 6.35 | 1.06 |
| LAMC1 | 82.58 | 66.4 | 1.24 | 8.57 | 7.88 | 1.09 |
| LTBP3 | 33.1 | 26.63 | 1.24 | 8.07 | 7.67 | 1.05 |
| MCAM | 120.01 | 96.89 | 1.24 | 7.77 | 7.19 | 1.08 |
| COL1A1 | 3.2 | 2.58 | 1.24 | 6.85 | 6.14 | 1.12 |
| VCAN | 22.23 | 18.08 | 1.23 | 9.32 | 8.84 | 1.05 |
| F3 | 389 | 316.27 | 1.23 | 8.63 | 8.18 | 1.05 |
| GALNT2 | 20.14 | 16.41 | 1.23 | 7.18 | 6.75 | 1.06 |
| DCHS1 | 4.16 | 3.39 | 1.23 | 6.91 | 6.39 | 1.08 |
| MFGE8 | 27.17 | 22.15 | 1.23 | 6.35 | 6.05 | 1.05 |
| CHL1 | 5.81 | 4.74 | 1.22 | 8.91 | 8.34 | 1.07 |
| ADAMTS1 | 32 | 26.17 | 1.22 | 6.86 | 6.22 | 1.1 |
| ADAMTS3 | 4.42 | 3.63 | 1.22 | 5.55 | 4.88 | 1.14 |
| PXDN | 75.89 | 62.36 | 1.22 | 8.34 | 7.55 | 1.1 |
| HGSNAT | 8.24 | 6.78 | 1.22 | 7.6 | 7.2 | 1.06 |
| CTGF | 187.35 | 154.27 | 1.21 | 8.55 | 8.14 | 1.05 |
| FBN1 | 34.94 | 28.78 | 1.21 | 6.51 | 6.1 | 1.07 |
| SDF4 | 62.39 | 51.4 | 1.21 | 8.04 | 7.62 | 1.06 |
| AASS | 12.19 | 10.05 | 1.21 | 5.74 | 5.42 | 1.06 |
| TTC28 | 12.45 | 10.33 | 1.21 | 6.71 | 6.31 | 1.06 |
| CLCC1 | 6.66 | 5.54 | 1.2 | 6.13 | 5.76 | 1.07 |
| DPY19L4 | 7.27 | 6.06 | 1.2 | 7.35 | 6.92 | 1.06 |
| AKR1C3 | 9.17 | 11.33 | 0.81 | 6.2 | 6.7 | 0.93 |
| MRPL41 | 64.57 | 79.88 | 0.81 | 3.94 | 4.05 | 0.97 |
| TMEM14A | 23.04 | 29.63 | 0.78 | 9.01 | 9.22 | 0.98 |
| MAGEA1 | 73.98 | 95.41 | 0.78 | 3.81 | 3.91 | 0.98 |
| E2F2 | 2.71 | 3.5 | 0.77 | 3.72 | 3.81 | 0.98 |
| RAB3B | 2.44 | 3.21 | 0.76 | 4.14 | 4.25 | 0.97 |
| FGF13 | 1.15 | 1.53 | 0.76 | 5.64 | 6.13 | 0.92 |
| RPH3AL | 0.56 | 0.74 | 0.75 | 4.5 | 4.59 | 0.98 |
| MAGEB2 | 31.53 | 42.96 | 0.73 | 3.9 | 4 | 0.97 |
| KRT81 | 360.91 | 554.87 | 0.65 | 5.01 | 5.15 | 0.97 |
| PLA2G1B | 0.02 | 0.26 | 0.08 | 4.41 | 4.5 | 0.98 |

**Table S6. Venn diagram analysis on GO_BP and KEGG genesets enriched by treatment of EFNA1 and PDGFA, respectively.**

| **Shared Gensets** | **PDGFA treatment specific genesets** | **EFNA1 treatment specific genesets** |
| --- | --- | --- |
| cell migration | Axon guidance | glycosaminoglycan biosynthetic process |
| glycosaminoglycan metabolic process | cell-cell adhesion | positive regulation of extrinsic apoptotic signaling pathway |
| glycosaminoglycan catabolic process | inflammatory response |  |
| HTLV-I infection | positive regulation of cellular protein metabolic process | viral entry into host cell |
| TNF signaling pathway | collagen catabolic process | cholesterol metabolic process |
| semaphorin-plexin signaling pathway | negative regulation of cell migration | Metabolic pathways |
| Adipocytokine signaling pathway | endoderm formation | negative regulation of tumor necrosis factor production |
|  | positive regulation of axonogenesis |  |
|  | regulation of angiogenesis | Non-alcoholic fatty liver disease (NAFLD) |
|  | Insulin resistance | mitochondrial electron transport, NADH to ubiquinone |
|  | response to retinoic acid |  |
|  | regulation of cell proliferation | negative regulation of inclusion body assembly |
|  | cellular response to hypoxia | Glycosaminoglycan biosynthesis - chondroitin sulfate / dermatan sulfate |
|  | intrinsic apoptotic signaling pathway in response to endoplasmic reticulum stress |  |
|  | ATP metabolic process | Lysosome |
|  | skeletal system development | Oxidative phosphorylation |
|  | cholesterol biosynthetic process | Huntington's disease |
|  | Focal adhesion |  |
|  | positive regulation of fat cell differentiation |  |
|  | Legionellosis |  |
|  | cellular response to tumor necrosis factor |  |
|  | lung development |  |
|  | negative regulation of cell growth |  |
|  | cysteine biosynthetic process |  |
|  | Rheumatoid arthritis |  |
|  | positive regulation of apoptotic cell clearance |  |
|  | decidualization |  |
|  | artery morphogenesis |  |
|  | positive regulation of peptidyl-tyrosine phosphorylation |  |
|  | MAPK signaling pathway |  |
|  | retinoid metabolic process |  |
|  | extracellular matrix organization |  |
|  | positive regulation of angiogenesis |  |
|  | cellular response to hormone stimulus |  |
|  | long-chain fatty-acyl-CoA biosynthetic process |  |
|  | Osteoclast differentiation |  |
|  | negative regulation of axon extension involved in axon guidance |  |
|  | BMP signaling pathway |  |
|  | ECM-receptor interaction |  |
|  | cell adhesion |  |
|  | PI3K-Akt signaling pathway |  |
|  | positive regulation of NF-kappaB transcription factor activity |  |
|  | Protein digestion and absorption |  |
|  | response to mechanical stimulus |  |
|  | cellular amino acid biosynthetic process |  |
|  | hyaluronan biosynthetic process |  |
|  | cellular response to UV-B |  |
|  | response to calcium ion |  |
|  | response to cAMP |  |
|  | neural crest cell migration |  |
|  | response to hydrogen peroxide |  |
|  | positive regulation of cholesterol efflux |  |
|  | collagen metabolic process |  |
|  | positive regulation of axon extension |  |
|  | lung morphogenesis |  |
|  | collagen fibril organization |  |
|  | glutathione biosynthetic process |  |
|  | endothelial cell migration |  |
|  | positive regulation of cell migration |  |
|  | intrinsic apoptotic signaling pathway in response to DNA damage |  |
|  | cell motility |  |
|  | angiogenesis |  |
|  | response to hypoxia |  |
|  | regulation of transcription from RNA polymerase II promoter |  |
|  | regulation of cell adhesion |  |
|  | Glycine, serine and threonine metabolism |  |
|  | long-chain fatty acid metabolic process |  |
|  | response to cytokine |  |
